# Development and preliminary validation of the Sjögren’s Tool for Assessing Response (STAR): a consensual composite score for assessing treatment effect in primary Sjögren’s syndrome

**DOI:** 10.1136/annrheumdis-2021-222054

**Published:** 2022-04-07

**Authors:** Raphaele Seror, Gabriel Baron, Marine Camus, Divi Cornec, Elodie Perrodeau, Simon J Bowman, Michele Bombardieri, Hendrika Bootsma, Jacques-Eric Gottenberg, Benjamin Fisher, Wolfgang Hueber, Joel A van Roon, Valérie Devauchelle-Pensec, Peter Gergely, Xavier Mariette, Raphael Porcher, the NECESSITY WP5- STAR development working group, Suzanne Arends

**Affiliations:** 1 Paris-Saclay University, INSERM UMR1184: Centre for Immunology of Viral Infections and Autoimmune Diseases, Le Kremlin-Bicetre, France; 2 Rheumatology, Assistance Publique-Hôpitaux de Paris (AP-HP), Hôpitaux universitaires Paris-Sud - Hôpital Bicêtre, Le Kremlin-Bicêtre, France; 3 Assistance Publique Hôpitaux de Paris, Hôtel Dieu hospital, Paris, France; 4 Centre d'Epidémiologie Clinique, INSERM U1153, Faculté de Médecine, Université Paris Descartes, Paris, France; 5 Rhumatologie, CHU Brest, Brest, France; 6 Université de Brest, INSERM UMR 1227, LBAI, Brest, France; 7 Rheumatology, University Hospitals Birmingham, Birmingham, UK; 8 Rheumatology, Milton Keynes University Hospital, Milton Keynes, UK; 9 University of Birmingham, Birmingham, UK; 10 Experimental Medicine and Rheumatology, William Harvey Research Institute, Queen Mary University of London, London, UK; 11 Rheumatology and Clinical Immunology, University Medical Center Groningen, University of Groningen, Groningen, The Netherlands; 12 Rheumatology, University Hospital of Strasbourg, Strasbourg, France; 13 Université de Strasbourg, IBMC, CNRS, UPR3572, Strasbourg, France; 14 National Institute for Health Research (NIHR) Birmingham Biomedical Research Centre, Birmingham, UK; 15 Rheumatology Research Group, Institute of Inflammation and Ageing, University of Birmingham, Birmingham, UK; 16 Novartis Pharma, Basel, Switzerland; 17 Immunology, Rheumatology and Clinical Immunology, Center of Translational Immunology, University Medical Center Utrecht, Utrecht University, Utrecht, The Netherlands; 18 Novartis Institutes for BioMedical Research Basel, Basel, Switzerland

**Keywords:** Sjogren's syndrome, outcome assessment, health care, patient reported outcome measures

## Abstract

**Objective:**

To develop a composite responder index in primary Sjögren’s syndrome (pSS): the Sjögren’s Tool for Assessing Response (STAR).

**Methods:**

To develop STAR, the NECESSITY (New clinical endpoints in primary Sjögren’s syndrome: an interventional trial based on stratifying patients) consortium used data-driven methods based on nine randomised controlled trials (RCTs) and consensus techniques involving 78 experts and 20 patients. Based on reanalysis of rituximab trials and the literature, the Delphi panel identified a core set of domains with their respective outcome measures. STAR options combining these domains were proposed to the panel for selection and improvement. For each STAR option, sensitivity to change was estimated by the C-index in nine RCTs. Delphi rounds were run for selecting STAR. For the options remaining before the final vote, a meta-analysis of the RCTs was performed.

**Results:**

The Delphi panel identified five core domains (systemic activity, patient symptoms, lachrymal gland function, salivary gland function and biological parameters), and 227 STAR options combining these domains were selected to be tested for sensitivity to change. After two Delphi rounds, a meta-analysis of the 20 remaining options was performed. The candidate STAR was then selected by a final vote based on metrological properties and clinical relevance.

**Conclusion:**

The candidate STAR is a composite responder index that includes all main disease features in a single tool and is designed for use as a primary endpoint in pSS RCTs. The rigorous and consensual development process ensures its face and content validity. The candidate STAR showed good sensitivity to change and will be prospectively validated by the NECESSITY consortium in a dedicated RCT.

**Key messages**

**What is already known about this subject?**

- Today, there are still no Disease Modifying Anti Rheumatic Drug licensed for patients with primary Sjögren’s syndrome (pSS).
- One explanation to this is due to limitations of current outcome measures used as primary endpoints, for example, the high placebo response rate, evaluation of either the symptoms or the systemic activity, and important features not being assessed.

**What does this study add?**

- We herein developed a consensual composite endpoint, the Sjögren’s Tool for Assessing Response (STAR), using data-driven methods based on nine randomised controlled trials and consensus techniques based on the opinion of 78 experts and 20 patients.
- STAR aims to resolve the issues on current outcome measures in pSS and encompasses all disease features in a single tool.

**How might this impact on clinical practice or future developments?**

- STAR is intended for use in clinical trials as an efficacy endpoint and intends to become the reference standard outcome measure in pSS.

## Introduction

For decades, evidence-based therapy in primary Sjögren’s syndrome (pSS) has largely been based on sicca features or patient-reported outcomes (PROs). Over the past 20 years, work from an international consortium, supported by the European Alliance of Associations for Rheumatology (EULAR), has led to the development and validation of the consensual EULAR Sjögren’s Syndrome Disease Activity Index (ESSDAI) and EULAR Sjögren’s Syndrome Patient Reported Index (ESSPRI).^1–3^ Both have emerged as reference standards to measure systemic activity and patients’ symptoms, respectively.

Thus, ESSDAI has been used as a primary endpoint in recent randomised controlled trials (RCT) testing biologics, and for the first time in pSS four RCTs have met their primary endpoint.^4–7^ ESSDAI has shown promising capability to monitor changes in disease activity and assess therapeutic efficacy. Nonetheless, several trials failed to show improvement in ESSDAI,^8–10^ perhaps due to inefficacy of the drugs, but also potentially to the relatively high placebo response rates observed with ESSDAI. Also, the lack of efficacy may be explained by the absence of assessment of important features in ESSDAI, such as patients’ symptoms and glandular function.^11^ Recent RCTs showed that improvement in ESSDAI does not necessarily translate to improvement in PROs.^4–7 12^ Thus, used as a unique primary endpoint, ESSDAI does not capture all important disease features. These limitations are inherent to scale constructs and highlight the need for a composite endpoint able to assess the disease globally.^13^

The NECESSITY (New clinical endpoints in primary Sjögren’s syndrome: an interventional trial based on stratifying patients) consortium (https://www.necessity-h2020.eu/) includes pSS experts from academia, pharmaceutical industry and patient groups formed to develop a new composite responder index, the Sjögren’s Tool for Assessing Response (STAR). STAR aims to resolve the issues on current outcome measures in pSS and is intended for use in clinical trials as an efficacy endpoint. We herein report its development process.

## Methods

**Figure 1:**
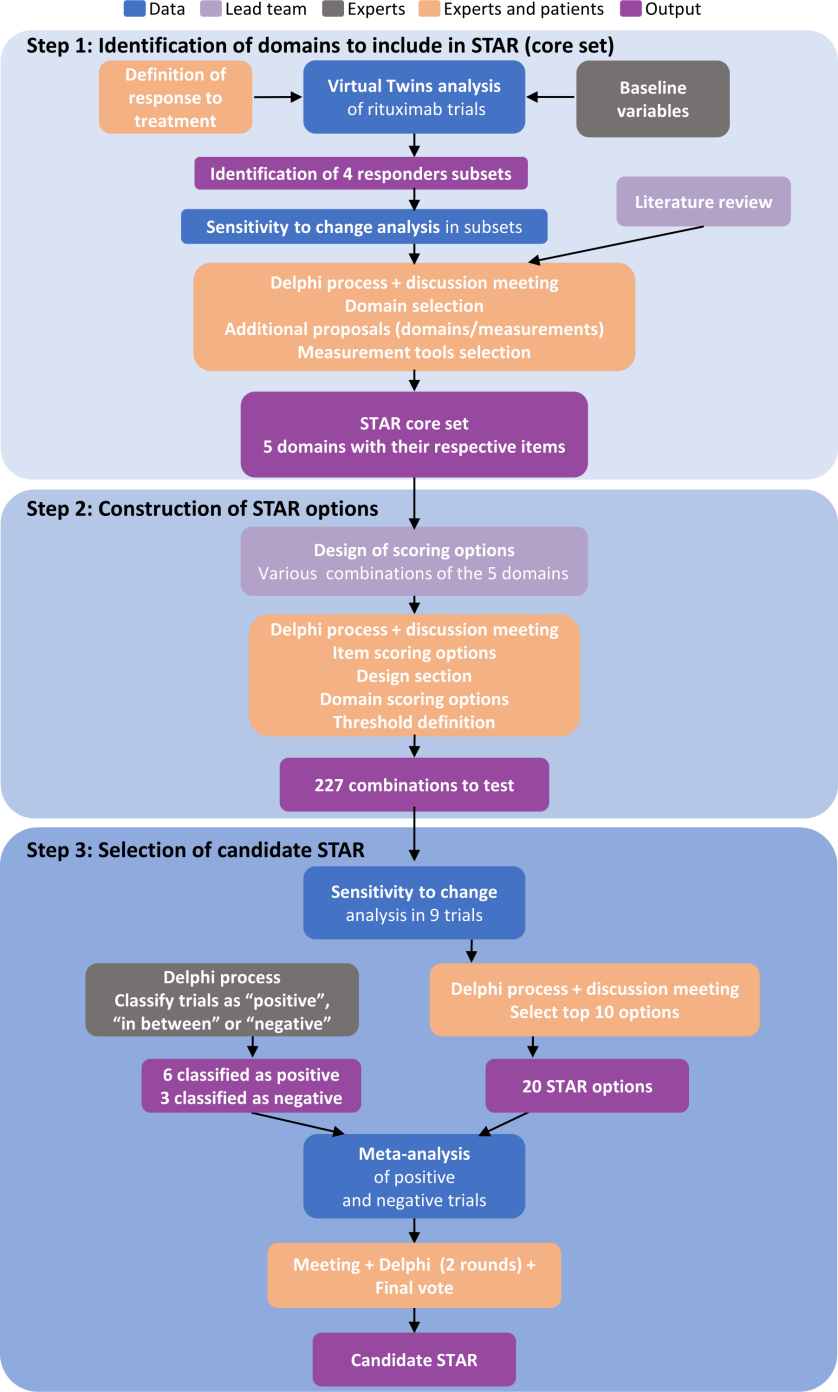
STAR development process. STAR, Sjögren’s Tool for Assessing Response.

The development and preliminary retrospective validation of STAR followed the OMERACT (Outcome Measures in Rheumatology) guidelines^14^ and consisted of three steps (figure 1), combining data-driven methods from nine RCTs (table 1) and consensus methods. The Delphi panel was formed by 78 pSS international experts (57 clinicians, 21 scientists) and 20 patients with pSS (online supplemental 1).

10.1136/annrheumdis-2021-222054.supp1 Supplementary data

**Table 1:** Description of the nine randomised controlled trials used for the development of STAR and their classification by the expert panel

Drug, first author, year	n	Primary endpoint	Visit used to compute STAR	Key secondary endpoints	Classification by experts
Abatacept (ASAP-III), van Nimwegen, 2020^8^	80	*Not met:* Change in ESSDAI at W24. Adjusted mean difference between groups at W24: −1.3 (95% CI −4.1 to 1.6), p=0.385.	W24	*No difference:* ESSPRI, PhGA, PatGA, OSS scores; UWSF, TBUT, Schirmer’s test (average in both eyes), ocular and oral dryness NRS values; C3/C4 level at W24. *Significant improvement:* IgG: adjusted mean difference between groups at W24: −0.1 (95% CI −0.2 to −0.01), p=0.028. RF: adjusted mean difference between groups at W24: −13.8 (95% CI −20.7 to −6.0), p<0.0001.	Positive (‘in between’).
Baminercept (BAMIN), St Clair, 2018^35^	52	*Not met:* Change in SWSF at W24. Baseline-adjusted mean change between groups at W24: −0.01 mL/min TTT vs 0.07 PBO, p=0.33.	W24	*No difference:* Change from baseline in ESSDAI, PhGA, PatGA, OSS scores; UWSF, dryness and fatigue VAS, Schirmer’s test values.	Negative.
Hydroxychloroquine (JOQUER), Gottenberg, 2014^36^	120	*Not met:* 30% reduction in 2 of 3 VAS (dryness, pain, fatigue) at W24. Percentage of responders, between-group difference at W24: 17.9% TTT vs 17.2% PBO; OR 1.01 (95% CI 0.37 to 2.78), p=0.98 (after multiple imputations).	W24	*No difference:* Change from baseline to W24 in ESSPRI, ESSDAI, PhGA scores; Schirmer’s test, UWSF values.	Negative.
Hydroxychloroquine and leflunomide (RepurpSS), van der Heijden, 2020^6^	29	*Met:* Change in ESSDAI at W24. Baseline-adjusted mean between-group difference at W24: −4.35 points (95% CI −7.45 to −1.25), p=0.0078.	W24	*No difference:* ESSPRI, PatGA scores; NRS oral and ocular dryness, Schirmer’s test, and C3/C4 levels at W24. *Significant improvement:* PhGA: baseline-adjusted mean between-group difference at W24: effect size −15.2 (95% CI −29.96 to −1.08), p=0.036. UWSF: baseline-adjusted mean between-group difference at W24: effect size: 10.57 (95% CI 2.21 to 18.93), p=0.014. Serum IgG: baseline-adjusted mean between-group difference at W24: effect size −3.32 (95% CI −5.28 to −1.37), p=0.0013. IgM-RF: baseline-adjusted mean between-group difference at W24: effect size: −0.59 (95% CI −1.06 to −0.12), p=0.017.	Positive.
Ianalumab (anti-BAFFR), Dörner, 2019^37^	27	*Not met:* Change in ESSDAI at W12 (combined as well as in individual dose groups).	W24	*Significant improvement:* ESSPRI (repeated measurement model). 10 mg/kg group: At W12: −1.55 points (95% CI 0.03 to 3.08). At W24: −1.92 points (95% CI 0.33 to 3.52). PatGA and PhGA.	Positive.
Iscalimab (anti-CD40), Fisher, 2020^5^	Total=44; in cohort 2=32	*Met for cohort 2 only (10 mg/kg intravenously):* Change in ESSDAI at W12. Baseline-adjusted between-group difference at W12: −5.21 points (95% CI 0.96 to 9.46), p=0.0090, one-sided.	W12	In cohort 2 only: *Significant improvement:* PhGA: between-group difference at W12: −12.16 (2.38 to 21.94). *Non-significant improvement:* ESSPRI: between-group difference at W12: −0.95 (−0.50 to 2.41). PatGA: between-group difference at W12: −8.14 (−10.39 to 26.67). UWSF: between-group difference at W12: 0.04 mL/min (−0.03 to 0.10). Schirmer’s test: between-group difference at W12: +8.06 mm (−1.37 to 17.50) for the left eye; +9.07 mm (−4.61 to 22.75) for the right eye.	Positive.
Rituximab (TRACTISS), Bowman, 2017^16^	133	*Not met:* 30% reduction in fatigue or oral dryness at W48. OR for RTX vs PBO=1.13 (95% CI 0.50 to 2.55), p=0.76. Baseline-adjusted absolute difference in response rates at W48: OR for RTX vs PBO=1.7 (95% CI −16.5 to 19.1), p=0.84.	W48	*No difference:* ESSPRI, ESSDAI scores, oral and ocular VAS values at W48. *Significant improvement:* UWSF: between-group difference at W48: OR 1.71 (95% CI 1.23 to 2.37), p=0.0015.	Positive (‘in between’).
Rituximab (TEARS) Devauchelle-Pensec, 2014^15^	120	*Not met:* 30 mm improvement in at least 2 of 4 VAS (PatGA, pain, fatigue, dryness). Percentage points between-group difference at W24: OR for RTX vs PBO: 1.0 (95% CI −16.7 to 18.7), p=0.91.	W24	*No difference:* ESSDAI score, PhGA, UWSF, Schirmer’s test values. *Significant improvement:* IgG level: between-group difference at W24: 1.2 g/L (95% CI 0.4 to 2.0), p=0.003.	Positive (‘in between’).
Tocilizumab (ETAP), Felten, 2020^10^	110	*Not met:* Percentage of responders at W24 defined as (1) decrease of ≥3 points in ESSDAI, (2) no occurrence of moderate or severe activity in any new domain of the ESSDAI compared with enrolment and (3) absence of worsening in PhGA. Percentage of responders: 54.2% TTT (41.3; 66.7%) vs 62.1% PBO (49.0; 74.1%), OR 1.6 (95% CI 0.3 to 3.3).	W12	*No difference:* ESSDAI score at W24 and change from baseline, ESSPRI score at W24 and change from baseline, UWSF value.	Negative.

ASAP-III, Abatacept Sjögren Active Patients Phase III Study; BAFFR, B-cell activating factor receptor; BAMIN, Baminercept; ESSDAI, EULAR Sjögren's Syndrome Disease Activity Index; ESSPRI, EULAR Sjögren's Syndrome Patient Reported Index; ETAP, Efficacy of TocilizumAb in Primary Sjögren’s syndrome; EULAR, European Alliance of Associations for Rheumatology; JOQUER, Randomized Evaluation of Hydroxychloroquine in Primary Sjogren’s Syndrome; n, number of participants; NRS, numeric rating scale; OSS, ocular staining score; PatGA, patient global assessment; PBO, placebo; PhGA, physician global assessment; RepurpSS, Leflunomide–hydroxychloroquine combination therapy in patients with primary Sjögren's syndrome; RF, rheumatoid factor; RTX, rituximab; STAR, Sjögren’s Tool for Assessing Response; SWSF, Stimulated Whole Salivary Flow; TBUT, Tear Break Up Time; TEARS, Tolerance and EfficAcy of Rituximab in primary Sjögren syndrome; TRACTISS, TRial of Anti-B-Cell Therapy In patients with primary Sjögren’s Syndrome; TTT, treatment; UWSF, unstimulated whole salivary flow; VAS, Visual Analogue Scale; W, duration in weeks.

### Step 1: identification of the STAR core set

This step aimed to select the core set of domains of relevance in assessing treatment response in pSS and the measurement tool and definition of response for each domain.

We used data from two rituximab trials because, although they failed to demonstrate treatment efficacy in their primary endpoint relying on PROs, clinical experience suggests that rituximab should work in at least some patients and for some endpoints.^15–17^ When only a portion of patients respond to the treatment, it might preclude the identification of an average treatment effect in the whole population. Many statistical methods exist to maximise the chances of detecting parameters that show differential change between active and placebo arms. We here used the virtual twins approach, which identifies subgroups with enhanced probability of response based on their baseline characteristics and which estimates the treatment effect in each subgroup while correcting for optimism due to the data-driven process.^18^

#### Identification of subsets of responders

The panellists first agreed, based on expertise, literature and patient feedback, on baseline variables to include in the analyses (ie, main pSS characteristics suspected to be associated with response to treatment), and on the definitions of response to treatment, based on existing outcome measures in pSS and validated cut-offs.

Virtual twins regression trees were computed for each definition of response and each set of baseline variables. Responder subsets (ie, a branch of virtual twins analysis) were selected by the lead team based on statistical criteria (a relative risk of response to treatment vs placebo notably higher than in the whole population and a sufficient number of patients (≥60) for statistical power) and clinical relevance (subset identified by a definition of response including both physician and PROs).

#### Identification of items sensitive to change

The items most sensitive to change were identified based on their effect size (ES; with their 95% CI) for the between-group difference of change in score from baseline to week 24 and in score at week 24 in each responder subset and the whole population. The ES for the difference between groups was assessed by the Cohen’s d measure, assuming a pooled SD.^19^ CIs were estimated using the non-centrality parameter approach. This method searches for the best non-central parameter (NCP) of the non-central t distribution for the desired tail probabilities, and these NCPs are then converted to the corresponding ES.^20^ The larger the ES, the greater the sensitivity to change.^21^ ES values are commonly considered large (>0.8), moderate (0.5–0.8) or small (<0.5). The following outcomes were analysed: specific scores (ESSDAI, ESSPRI, and physician and patient global assessment), dryness (global, oral and ocular), pain and fatigue Visual Analogue Scale, glandular function (Schirmer’s test and salivary flow), and biological variables (β2 globulin, serum IgG, γ-globulin, erythrocyte sedimentation rate, rheumatoid factor (RF) and C4 complement).

#### Selection of domains, items and definition of response

The results of the analyses on sensitivity to change and relevant literature review were presented to the Delphi panel. The scoping review of the literature on outcome measures in pSS will be published elsewhere. Based on these data and on clinical experience, the Delphi panellists were asked to rate the importance of measuring each outcome in the context of assessing treatment response in clinical trials (from not important (1–3) to critical (7–9) on a 9-point Likert scale) and to provide comments and suggest new domains or measurements. Items scored as critical (score ≥7) by ≥50% of the panellists were selected and were defined as the domains to include in STAR. Several items and definitions of response were selected in each domain.

### Step 2: construction of STAR options

The lead team prepared the drafts of the STAR options, combining the items and definitions of response identified previously. These draft options, along with the recently developed concise Composite of Relevant Endpoints for Sjögren’s Syndrome (CRESS),^22^ were presented to the panellists to select by vote which designs will be analysed in the next step. They could also make suggestions of combinations and alternate measurement tools or thresholds. Designs with ≥50% of votes, modified as per experts’ suggestions, were selected.

### Step 3: evaluation of sensitivity to change of STAR options and selection of the candidate STAR

This phase aimed at selecting the candidate STAR and relied on analysis of nine RCTs completed at the time of analysis (table 1).

#### Analysis of sensitivity to change of STAR options

The responder rate in each group for binary options (or the mean score for continuous options) was calculated for each STAR option in each RCT. Sensitivity to change was estimated using the concordance (C) index,^23^ which is similar to the area under the curve of the receiver operating characteristics curve for a binary outcome. It ranges from 0 to 1 and is interpreted as follows: 1, perfectly discriminant; 0.5–1, more discriminant than random; and <0.5, worse than random.

#### Voting for top 10

These analyses along with explanations on data interpretation were presented to the expert panel. They were asked to vote for their top 10 options. During a follow-up meeting, the results of the vote were discussed to consensually select the options for the next step.

#### Meta-analysis of the selected options

To better appraise the sensitivity to change of the remaining STAR options, the Delphi panel decided to perform a meta-analysis of the nine RCTs. The Delphi panel voted on which trials they considered positive, negative or ‘in between’ with regard to primary but also key secondary endpoints. A study that failed to meet its primary outcome was considered ‘in between’ if the experts agreed that there was sufficient signal of benefit in the secondary outcomes. Meta-analyses were run for 'positive' and ‘in between’ trials together in which positive results were expected, and separately for negative trials in which no difference between groups was expected.

For binary outcomes, meta-analyses were run using the Mantel-Haenszel method with the Paule-Mandel estimator for *τ* ^2^, Q-profile method for the CI of *τ* ^2^and *τ*, and continuity correction of 0.5 in studies with zero cell frequencies.^24^ For continuous outcomes, the inverse variance method was used with the Paule-Mandel estimator for *τ* ^2^, Q-profile method for the CI of *τ* ^2^and *τ*, and Hedges’ g.

For binary scores, the treatment effect was expressed as OR, where 1 or below indicates absence of any effect, while above 1 favours the experimental treatment. For continuous scores, the treatment effect was expressed as standardised mean difference, where 0 indicates absence of any effect, while above 0 favours the experimental treatment. Consequently, a STAR option that is sensitive and specific to change should have a treatment effect close to the null effect for the negative trials and as far from the null effect for the positive trials.

#### Voting for top 3

The results of the meta-analyses were shared with the Delphi panel, who then voted for their top 3 options. During a follow-up meeting, the results were discussed to consensually select the options for the next step.

#### Voting for the candidate STAR

A final vote was run to select the candidate STAR based on clinical relevance.

### Patient involvement

The NECESSITY Patient Advisory Group (PAG) representatives were involved in all steps and participated in every discussion meeting. Other patients contacted by the PAG representatives participated anonymously in the development of STAR (steps 1 and 3). Only PAG representatives participated in step 2 because this exercise required technical knowledge of endpoint construction. The background information provided in each survey was tailored to the patients.

## Results

### Step 1: identification of the STAR core set

#### Identification of subsets of responders

The Delphi panel selected two sets of baseline variables for analyses, one with ESSDAI and ESSPRI total scores (set 1) and one with their subscales/domains (set 2) (online supplemental 2), and proposed 14 definitions of response to treatment (online supplemental 3). Virtual twins regression trees were computed and the lead team selected four responder subsets (online supplemental 4).

#### Identification of items sensitive to change

**Figure 2:**
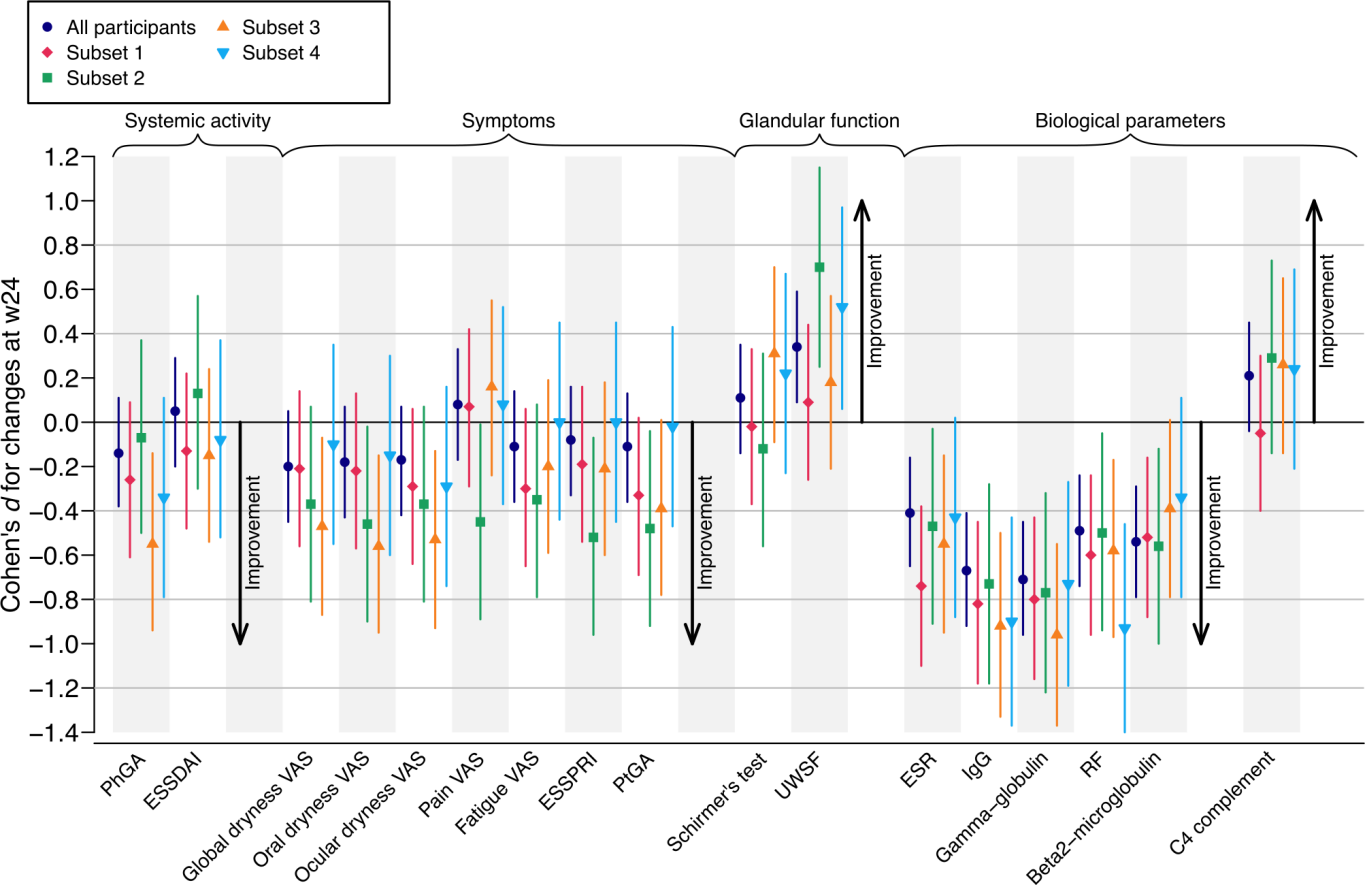
Sensitivity to change of each individual outcome in the combined analysis of the TEARS and TRACTISS rituximab trials. Sensitivity to change is represented by the Cohen’s effect size and the 95% CI. Analyses relied on a combined analysis of data from TEARS and TRACTISS rituximab trials. Cohen’s effect size and 95% CI for the standardised difference in mean change from baseline to W24 were computed for each outcome in the four responder subsets and in the whole population of the two trials. ESR, erythrocyte sedimentation rate; ESSDAI, EULAR Sjögren’s Syndrome Disease Activity Index; ESSPRI, EULAR Sjögren’s Syndrome Patient Reported Index; PtGA, patient global assessment; PhGA, physician global assessment; RF, rheumatoid factor; TEARS, Tolerance and Efficacy of Rituximab in primary Sjögren Syndrome; TRACTISS, TRial of Anti-B Cell Therapy In patients with primary Sjögren Syndrome; UWSF, unstimulated whole salivary flow; VAS, Visual Analogue Scale; W, duration in weeks.

Analysis of sensitivity to change of each outcome revealed that some outcomes improved significantly better in the rituximab arms compared with the placebo arms in at least one responder subset and/or in the whole population (figure 2): (1) among PROs, dryness (overall, oral or ocular) and ESSPRI; (2) among objective dryness measures, unstimulated whole salivary flow (UWSF) but not Schirmer’s test; and (3) among biological markers, serum IgG, γ-globulin and RF levels. By contrast, systemic scores did not improve in any subset, except for physician global assessment in subset 3. The results were similar when analysing the ES for between-group differences of change in score from baseline to week 24 (figure 2) or the final value at week 24 (online supplemental 5).

#### Selection of domains, items and definition of response

Five domains were identified by the Delphi panel: systemic activity, patient symptoms, lachrymal gland function, salivary gland function and biological parameters. No other domain was suggested.

For each domain, voting results, as well as clinical relevance, feasibility at clinical sites, and acceptability for patients and regulatory agencies, were considered when selecting the measurement tools. Thus, the Delphi panel selected either one or two measurement tools per domain (online supplemental 6 and 7). For the systemic domain, clinESSDAI was preferred to ESSDAI to avoid redundant recording of the biological parameter.^25^ For each glandular domain, two measurement tools were included to ensure the score could be calculated regardless of equipment availability at clinical sites.

### Step 2: construction of STAR options

Various designs for STAR were prepared by the lead team (table 2). The designs were inspired by the Disease Activity Score 28,^26^ Systemic Lupus Responder Index,^27^ American College of Rheumatology response criteria^28^ and by the recently developed clinical CRESS.^22^ Various cut-off values were proposed for each measurement. In some designs, due to their importance, systemic activity and PROs were defined as major domains that must improve to meet the definition of a responder. A total of 227 options were selected after voting and discussion meeting (online supplemental 8).

**Table 2:** Description of the STAR design proposed (step 2) and tested for sensitivity to change (step 3)

STAR’s proposed designs	Binary/continuous	Major domain/weight	Definition of response
Design 1 (DAS-28-like)	Continuous	No major domain. Need to define a weight for each component (using PatGA or PhGA as gold standard).	Total score will be the sum of the weighted domains. Threshold of response to be further defined.
Design 2 (SRI-like)	Binary	2 major domains (systemic activity *and* PROs). 3 minor domains.	Improvement of one of the major domains and no worsening of the other domains.
	Binary	Only 1 major domain (systemic activity *or* PROs depending on the primary objective of the study/drug). 4 minor domains.	Improvement of the target major domain and no worsening of the other domains.
Design 3A	Binary	2 major domains (systemic activity and PROs). 3 minor domains.	Improvement in ≥3 of 5 domains with improvement in at least 1 major domain.
Design 3B	Binary or continuous	3 points for major domains (systemic activity and PROs). 1 point for minor domain.	The score is the sum of the domains. For binary option: response is defined by a threshold of response (tested from 4 to 9). For continuous options: the total score is compared between groups.
Design 4 (ACR-like)	Binary	2 major domains (systemic activity and PROs). 3 minor domains.	Improvement (of xx%, from 10% to 70%) in ≥3 of 5 domains, including at least 1 major domain.
CRESS^22^	Binary	None.	Improvement in ≥3 of 5 domains.

ACR, American College of Rheumatology; CRESS, Composite of Relevant Endpoints for Sjögren's Syndrome; DAS-28, Disease Activity Score 28; PatGA, patient global assessment; PhGA, physician global assessment; PRO, patient-reported outcome; SRI, Systemic Lupus Responder Index; STAR, Sjögren’s Tool for Assessing Response.

### Step 3: evaluation of sensitivity to change of STAR options and selection of the candidate STAR

Analysis of sensitivity to change was run for the 227 options in the nine RCTs (online supplemental 9). Options in STAR design 1 were rejected because it was not possible to obtain a stable estimation of domain weights to construct a score. Of the 225 remaining options, 189 were never selected and were rejected, and 16 additional options, found to be redundant or less clinically relevant than the others, were rejected during the follow-up meeting. Consequently, 20 options moved to the next step.

**Figure 3:**
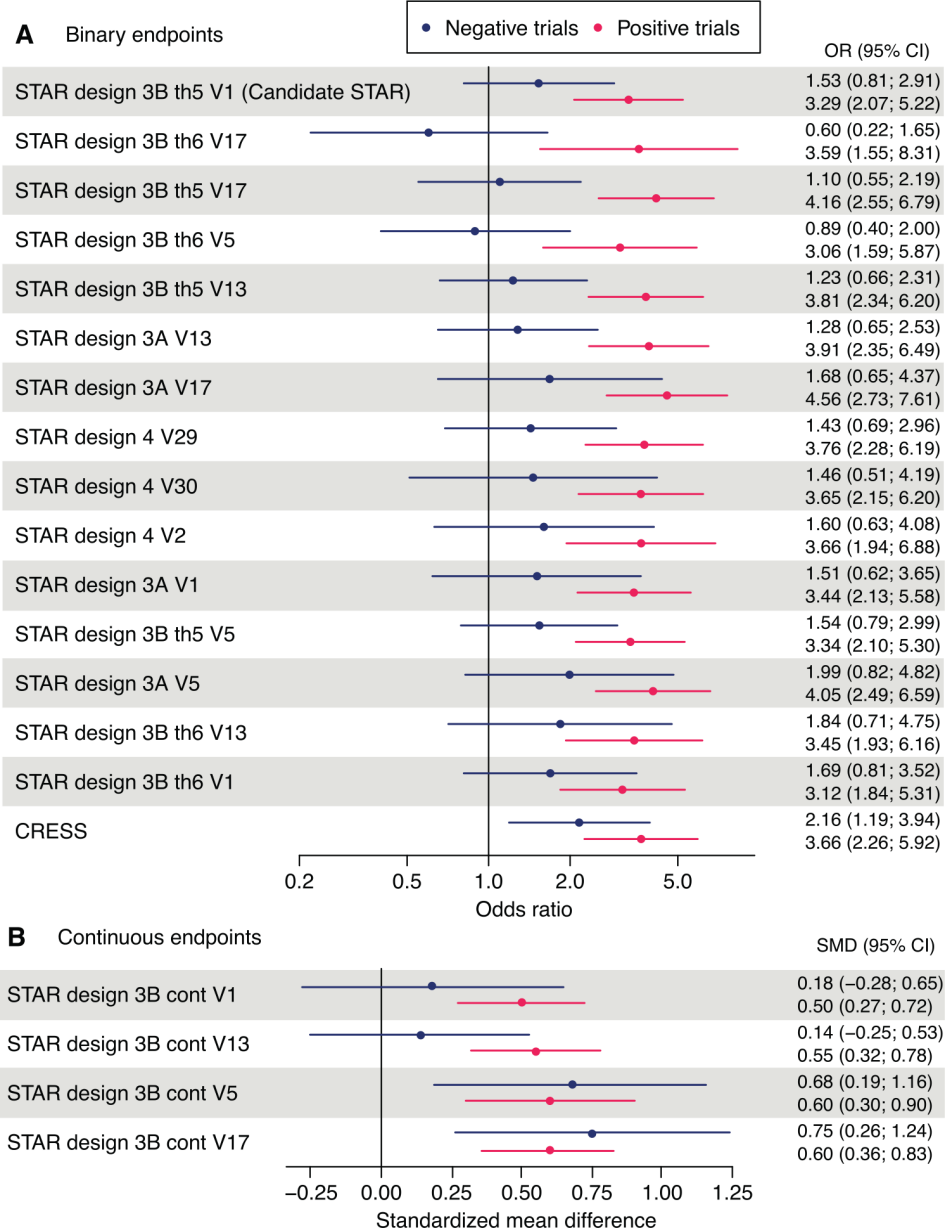
Results of meta-analyses on six studies considered positive and three considered negative by the experts. Meta-analyses were performed for the 20 STAR options that reach the final step and are presented for binary endpoints (panel A) et continuous endpoints (panel B). Interpretation: a score that is sensitive to change and specific to the treatment should have a treatment effect close to the null effect in the negative trials and as far as possible from the null effect in the positive trials. cont, continuous; CRESS, Composite of Relevant Endpoints for Sjögren’s Syndrome; SMD, standardised mean difference; STAR, Sjögren’s Tool for Assessing Response; th5, threshold 5; th6, threshold 6; V, version.

Based on the panellists’ classification of RCTs (online supplemental 10), meta-analyses were computed separately for trials considered 'positive' and or trials considered 'negative' by the experts (figure 3) to allow for comparison of sensitivity and specificity to change, respectively. Based on these results, the panellists voted for their top 3 options. Five options not selected by any panellist were not included in the final vote.

During the follow-up meeting, the panellists agreed that the selection of the candidate STAR from the remaining 15 options should be based on clinical relevance. The rationale for selection was as follows. A decrease in clinESSDAI was preferred to a set score (<5 points) at the final evaluation to avoid defining this domain as responder while the score did not change from baseline in patients with baseline low activity. ESSPRI was preferred to individual dryness scales because it is a validated score. The panellists selected the published minimal clinically important difference (MCID) as the response cut-off for clinESSDAI (≥3 points) and ESSPRI (≥1 point). Finally, the experts rejected the ‘no worsening’ clause because there is no published consensual definition for worsening of these outcomes and the options with this clause did not show better discriminative capacity (table 3). Finally, since the other 19 options (online supplemental 11) had good psychometric properties, they will be evaluated as exploratory endpoints in the NECESSITY clinical trial (EudraCT no: 2019-002470-32; online supplemental 12).

**Table 3:** Candidate STAR

Domain	Point	Definition of response
Systemic activity	3	Decrease of ≥3 in clinESSDAI.
Patient-reported outcome	3	Decrease of ≥1 point or ≥15% in ESSPRI.
Lachrymal gland function (assessed by Schirmer’s test or ocular staining score)	1	Schirmer’s test: If abnormal score at baseline: increase ≥5 mm from baseline. If normal score at baseline: no change to abnormal. *Or* Ocular staining score: If abnormal score at baseline: decrease of ≥2 points from baseline. If normal score at baseline: no change to abnormal.
Salivary gland function (assessed by unstimulated whole salivary flow or ultrasound)	1	Unstimulated whole salivary flow: If score is >0 at baseline: increase of ≥25% from baseline. If score is 0 at baseline: any increase from baseline. *Or* Ultrasound: Decrease of ≥25% in total Hocevar score from baseline.
Biological (assessed by serum IgG or RF level)	1	Serum IgG level: decrease of ≥10%. *Or* RF level: decrease of ≥25%.
Candidate STAR responder		≥5 points

For ocular tests, Schirmer’s test should be performed without anaesthesia and is considered abnormal if <5 mm. Ocular staining score is considered abnormal if score is ≥3. The mean of both eyes was used for calculation.

Total RF or RF-IgM was measured in IU/mL.

For unstimulated whole salivary flow, we recommend establishing an SOP for each future trial using STAR. The SOP should specify if the collection should be done over 5 or 15 min (both are possible but one option should be selected for each trial and applied to all patients), and should specify that patients should no eat, drink or smoke for 60 min before the collection, should not take secretagogue morning dose, and should perform the collection in the morning and at a fixed time.

ESSDAI, EULAR Sjögren's Syndrome Disease Activity Index; ESSPRI, EULAR Sjögren's Syndrome Patient Reported Index; RF, rheumatoid factor; SOP, standard operating procedures; STAR, Sjögren’s Tool for Assessing Response.

## Discussion

The NECESSITY consortium, supported by an international panel of pSS experts, scientists, methodologists and patients, developed a consensual single tool for pSS that globally assesses all disease features and for use as an efficacy endpoint in RCTs: the composite responder index STAR. STAR fulfils the truth, discrimination and feasibility criteria recommended by OMERACT. The strength of our work relies on a rigorous process combining both consensus techniques based on the opinion of a large panel and data-driven methods generated from nine trials. In the analyses performed separately for trials considered negative and positive by the expert consensus, our study demonstrated that the candidate STAR is able to show treatment efficacy in positive trials and did not erroneously detect significant between-arm differences in trials considered negative, as did some alternate options (figure 3).

Designing a primary endpoint in pSS is challenging due to the wide spectrum of disease features and the great heterogeneity and complexity of signs and symptoms. Major changes in RCT design recently conducted to adoption of ESSDAI as primary outcome and allowed, for the first time, demonstration of treatment efficacy (table 1). However, these trials suggested that other outcomes might also improve with treatment, such as ESSPRI, UWSF and biological components (IgG and RF levels). However, recent trials focused on patients with moderate to high systemic disease activity, excluding a large proportion of patients with no systemic complications but with high symptom burden. In pSS, low quality of life is mainly driven by PROs rather than systemic activity^29^; also, these two domains poorly correlate.^11 30 31^ STAR can evaluate treatment response in the full spectrum of patients with pSS, including those with low systemic activity but high burden of symptoms, for whom there remains an important unmet need. Effectively, to avoid the pitfalls of a data-driven process relying on a single trial, the development of the candidate STAR relied on nine trials, some of which included patients with low systemic disease activity and having various timepoints of evaluation (12–48 weeks, but 24 weeks in most cases). A recent important initiative from a group in the Netherlands, also a NECESSITY partner, proposed the CRESS based on reanalysis of the ASAP-III (Abatacept Sjögren Active Patients Phase III Study) trial.^8 22^ The CRESS, similar to STAR, also includes the same five domains, confirming their clinical relevance in the global assessment of pSS. However, STAR has defined two major domains, systemic activity and patient symptoms, and the definition of response requires improvement of at least one. Thus, unlike CRESS, STAR requires improvement of PROs in patients with low systemic activity. Also, in negative trials, where no difference between arms is expected, the candidate STAR, accurately, did not detect any difference between arms, where other options such as the concise CRESS did (figure 3). STAR also includes improvement of glandular function using simple and validated measures, that is, Schirmer’s test, sicca ocular staining score (OSS)^32^ and UWSF, but also includes salivary gland ultrasound, leaving the door open to more sophisticated tests to evaluate these domains in the future. Lastly, and although they do not reflect patients’ perceived disease burden, the experts decided to include IgG and RF levels because they considered, whatever the mechanism of action of the drug, a therapeutic goal to decrease the levels of these biomarkers, signs of activity (IgG) or predictive markers of lymphoma (RF).^33 34^

Nevertheless, our study has some limitations. The main issue is circular thinking since pSS experts may be tempted to define a patient as a responder or a non-responder or a trial as positive or negative based on pre-existing indexes. This may give high weight to previous indexes, leaving little room for very innovative items, which by definition were not included in previous RCTs and cannot be evaluated at this stage. Nevertheless, theses definitions relied on a high level of consensus (online supplemental 10) after evaluation of multiple independent RCTs. Finally, in most of the trials, OSS, ultrasound data and RF levels were not available and thus the impact of these outcomes on STAR response cannot be evaluated at this stage.

The NECESSITY PAG strongly supports the STAR outcome (see letter of support in online supplemental 13). Recommendations from the European Medicines Agency (EMA) were sought through a scientific advice procedure, and the EMA has offered to publish on their website a letter of support for STAR (https://www.ema.europa.eu/en/documents/other/letter-support-sjogrens-tool-assessing-response-star_en.pdf). Also, additional steps are being worked on in collaboration with OMERACT to fulfil all requirements and for STAR to be formally endorsed.

Even though this process relied on a nearly never-equal number of experts and RCTs, further to the present retrospective validation, STAR has to be prospectively validated in an independent population in the NECESSITY RCT (online supplemental 12). The strength of this validation step is its evaluation of the psychometric properties of STAR, in particular its discriminant capacity in an interventional study where active and placebo arms will be compared. Also, patients will be stratified according to systemic activity, allowing the evaluation of the properties of STAR in any patient with pSS with either high systemic activity or high level of symptoms. We strongly encourage the use of the candidate STAR to evaluate its properties in diverse patient populations with treatments of various mechanisms of action to definitively validate STAR as a gold standard outcome measure for RCTs in pSS.

## Data Availability

No data are available. Data have been obtained and analysed in accordance with the NECESSITY consortium agreement and relevant data sharing agreements signed among the parties. They cannot be shared with other partners.
