# Supplementary material for: Development and preliminary validation of the Sjögren’s Tool for Assessing Response (STAR): a consensual composite score for assessing treatment effect in primary Sjögren’s syndrome
Source: Ann Rheum Dis. 2022 Apr 7;81(7):979–89. doi: 10.1136/annrheumdis-2021-222054 (PMC9209686; doi:10.1136/annrheumdis-2021-222054)
Supplement: Supplementary data [file annrheumdis-2021-222054supp001.pdf]

Development and preliminary validation of the Sjögren's Tool for Assessing Response (STAR): a consensual composite score for assessing treatment effect in primary Sjögren's syndrome

Supplements

Supplement 1: Delphi Panel: Characteristics of experts and patients who participated to the development of STAR 2

STEP 1: DEFINITION OF CORE SET

Supplement 2: Identification of responder subsets (Step 1): Baseline characteristics (variables) included in the virtual twins analysis for identifying subsets 8

Supplement 3: Identification of responder subsets (Step 1): Definitions of a response to treatment used in the virtual twins analysis for identifying subsets 9

Supplement 4: Identified responder subsets and their characteristics (Step 1) 10

Supplement 5: Identification of items sensitive to change (step 1): Effect size for the standardized difference in mean values at W24 of each outcome in the 4 responder subsets and the whole population of the two rituximab trials. 11

Supplement 6: Definition of core set of outcome measures: Experts and patients' scoring of items/domains (Step 1) 12

Supplement 7: Definition of core set of outcome measures (step 1): Results of patients and experts' vote and final selection 16

STEP 2: CONSTRUCTION OF STAR OPTIONS

Supplement 8: Construction of STAR options (step2): Description of options tested for sensitivity to change 19

STEP 3: EVALUATION OF SENSITIVITY TO CHANGE OF STAR OPTIONS AND SELECTION OF CANDIDATE STAR

Supplement 9: Sensitivity to change for STAR options: 22

Supplement 10: Classification of trials as 'positive' or 'negative': Results of vote 26

Supplement 11: Description of STAR alternate options 27

Supplement 12: Design of the NECESSITY trial 38

Supplement 13: Letter of support from the NECESSITY Patient Advisory Group 39

Supplement 1: Delphi Panel: Characteristics of experts and patients who participated to the development of STAR

Experts:

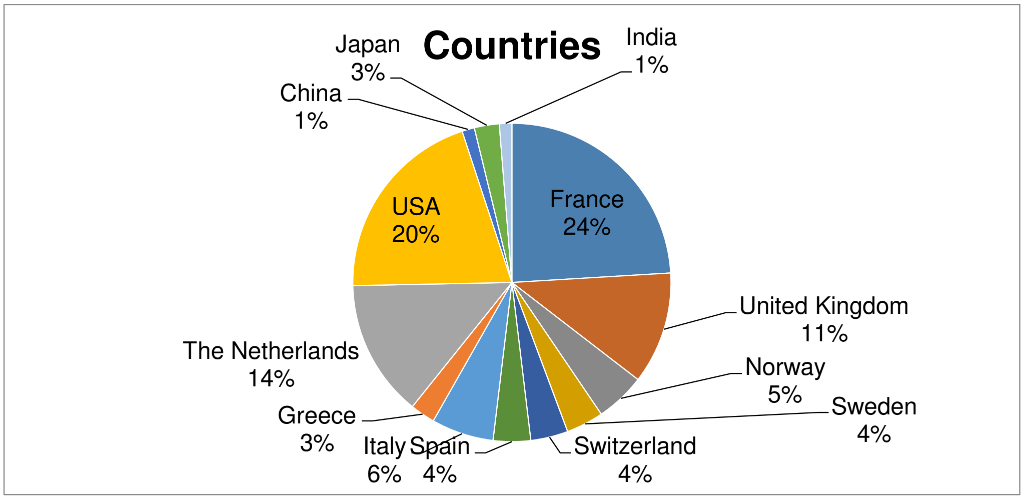

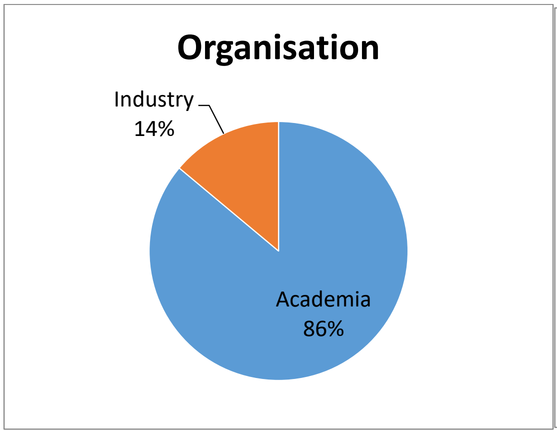

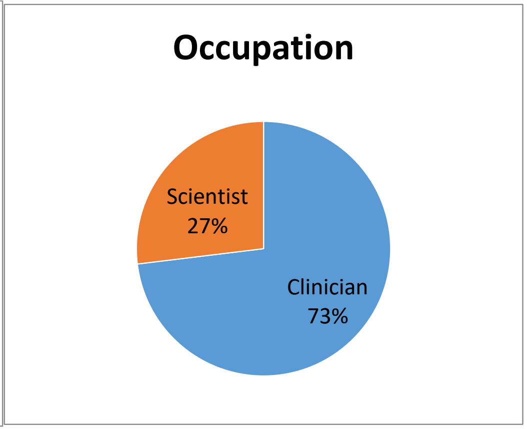

List of experts:

Name	Continent	Affiliation	Expertise	Status	Steps
Esen Karamursel Akpek	America	Johns Hopkins Medicines, USA	Clinician (ophthalmology)	External	1
Suzanne Arends	Europe	Universitair Medisch Centrum Groningen, Netherland	Scientist	NECESSITY partner	2-3
Alan Baer	America	Johns Hopkins Medicines, USA	Clinician (rheumatology)	External	1
Chiara Baldini	Europe	Università di Pisa, Italy	Clinician (immunology, rheumatology)	External	1, 3
Francesca Barone	Europe	University Hospital Birmingham, UK	Clinician (rheumatology)	NECESSITY partner	1
Elena Bartoloni	Europe	Università degli Studi di Perugia, Italy	Clinician (rheumatology)	External	1
Marí-Alfonso Begona	Europe	Corporació Sanitària Parc Taulí, Spain	Clinician (rheumatology)	External	1
Albin Björk	Europe	Karolinska Institute, Sweden	Clinician (rheumatology)	NECESSITY partner	3
Michele Bombardieri	Europe	Queen Mary University of London, UK	Clinician (rheumatology)	NECESSITY partner	1, 3
Hendrika Bootsma	Europe	Universitair Medisch Centrum Groningen, Netherland	Clinician (rheumatology)	NECESSITY partner	1-3
Simon Bowman	Europe	University Hospital Birmingham, UK	Clinician (rheumatology)	NECESSITY partner	1-3
Johan Brun	Europe	Haukeland University Hospital, Norway	Clinician (rheumatology)	External	1
Vatinee Bunya	America	Penn Medicines, USA	Clinician (ophthalmology)	External	1
Guillermo Carvajal Alegria	Europe	Brest hospital, France	Clinician (rheumatology)	NECESSITY partner	1
Wen-Hung Chen	America	GlaxoSmithKline, USA	Scientist (Senior Director, Specialty & Primary Care Head, Patient-Centered Outcomes)	NECESSITY partner	1-3
Laurent Chiche	Europe	Hôpital Européen Marseille, France	Clinician (rheumatology)	External	1
Kenneth Clark	Europe	GlaxoSmithKline, UK	Scientist (Senior Director, Clinical development)	NECESSITY partner	1-3
**Divi Cornec**	Europe	**Hôpital de la Cavale Blanche, Brest, France**	**Clinician (rheumatology)**	**NECESSITY partner, lead team**	**1-3**
Troy Daniels	America	University of California, San Francisco, USA	Clinician (dentistry)	External	1

3

Salvatore De Vita	Europe	University of Udine, Udine, Italy	Clinician (rheumatology)	NECESSITY partner	3
Liseth de Wolff	Europe	Universitair Medisch Centrum Groningen, Netherland	Scientist	NECESSITY partner	3
Konstantina Delli	Europe	Universitair Medisch Centrum Groningen, Netherland	Clinician (dentistry)	NECESSITY partner	1
Valérie Devauchelle-Pensec	Europe	Hôpital de la Cavale Blanche, Brest, France	Clinician (rheumatology)	NECESSITY partner	1, 3
Dewi Guellec	Europe	Hôpital de la Cavale Blanche, Brest, France	Clinician (rheumatology)	NECESSITY partner	1
Paul Emery	Europe	University of Leeds, UK	Clinician (rheumatology)	External	1
Jennifer Evans	America	Novartis, USA	Scientist (Associate Director, Global Regulatory Labeling)	NECESSITY partner	1, 2
Benjamin Fisher	Europe	University Hospital Birmingham, UK	Clinician (rheumatology)	NECESSITY partner	1-3
Robert Fox	America	Scripps Memorial Hospital La Jolla campus, USA	Clinician (rheumatology)	External	1
Stéphanie Galtier	Europe	Servier laboratories, France	Scientist (statistics)	NECESSITY partner	3
Saviana Gandolfo	Europe	Università degli Studi di Udine, Italy	Clinician (rheumatology)	NECESSITY partner	1, 2
Peter Gergely	Europe	Novartis Institutes for BioMedical Research, Switzerland	Scientist (Executive Director and Head of Rheumatology)	NECESSITY partner	1-3
Roberto Giacomelli	Europe	Università degli Studi dell-Aquila, Italy	Clinician (rheumatology)	External	1
John Gonzales	America	University of California, San Francisco, USA	Clinician (ophtalmology)	External	1
Jacques-Eric Gottenberg	Europe	Hôpital Universitaire Strasbourg, France	Clinician (rheumatology)	NECESSITY partner	1, 3
John Greenspan	America	University of California, San Francisco, USA	Scientist	External	1
Safae Hamkour	Europe	UMC Utrecht Universiteit, Netherland	Scientist	NECESSITY partner	1, 3
Dominik Hartl	Europe	Novartis Institutes for BioMedical Research, Switzerland	Clinician (Therapeutic Area Head Autoimmunity – Inflammation - Transplantation / Translational Medicine / Biomarkers), scientist	NECESSITY partner	1
Wolfgang Hueber	Europe	Novartis Institutes for BioMedical Research, Switzerland	Scientist(Translational Medicine / Autoimmunity)	NECESSITY partner	3

4

Malin V. Jonsson	Europe	University of Bergen, Norway	Scientist	NECESSITY partner	1
Roland Jonsson	Europe	University of Bergen, Norway	Scientist	NECESSITY partner	1
Frans Kroese	Europe	Universitair Medisch Centrum Groningen, Netherland	Clinician (rheumatology)	NECESSITY partner	2, 3
Aike Albert Kruize	Europe	Universitair Medisch Centrum Groningen, Netherland	Clinician (rheumatology)	NECESSITY partner	1
Laurence Laigle	Europe	Servier laboratories, France	Clinician (Director, Head of Biomarker development strategy, immuno-inflammatory diseases)	NECESSITY partner	1-3
Véronique Le Guern	Europe	Assistance Publique Hôpitaux de Paris, France	Clinician (rheumatology)	NECESSITY partner	1
Wen-Lin Luo	America	Novartis Pharmaceuticals Corporation, USA	Scientist (statistics)	NECESSITY partner	2
**Xavier Mariette**	Europe	**Assistance Publique Hôpitaux de Paris, France**	**Clinician (rheumatology)**	**NECESSITY partner, lead team**	**1-3**
Robert Moots	Europe	Liverpool University Hospitals NHS Foundation Trust, UK	Clinician (rheumatology)	External	1
Esther Mossel	Europe	Universitair Medisch Centrum Groningen, Netherland	Clinician (rheumatology)	NECESSITY partner	1
Wan-Fai Ng	Europe	Newcastle upon Tyne Hospitals NHS Foundation Trust, UK	Clinician (rheumatology)	NECESSITY partner	1-3
Susumu Nishiyama	Asia	Rheumatic Disease Centre, Kurashiki Medical Center, Kurashiki, Japan	Clinician (rheumatology)	External	1
Gaëtane Nocturne	Europe	Assistance Publique Hôpitaux de Paris, France	Clinician (rheumatology)	NECESSITY partner	1-3
Marleen Nys	America	Bristol Myers Squibb, USA	Scientist (statistics)	NECESSITY partner	3
Peter Olsson	Europe	Lund University, Sweden	Clinician (rheumatology)	External	1
Roald Omdal	Europe	University of Bergen, Norway	Clinician (rheumatology),	NECESSITY partner	1
Elodie Perrodeau	Europe	Assistance Publique Hôpitaux de Paris, France	Scientist (statistics)	NECESSITY partner	3
Jacques-Olivier Pers	Europe	Université de Bretagne Occidentale, France	Scientist	NECESSITY partner	1, 2
**Raphaël Porcher**	Europe	**Paris Université, France**	**Scientist (statistics)**	**NECESSITY partner**	**2, 3**

Elizabeth Price	Europe	Great Western Hospital, Swindon, UK	Clinician (rheumatology)	External	1
Manel Ramos-Casals	Europe	Hospital Clinic de Barcelona Institut Clinic de Medicina i Dermatologia, Barcelona, Spain	Clinician (rheumatology)	NECESSITY partner	1-3
Philippe Ravaud	Europe	Paris University, France	Clinician (rheumatology)	NECESSITY partner	3
Neelanjana Ray	America	Bristol Myers Squibb, USA	Scientist (Senior Director, Development Program Lead - Immunology, Research & Early Development)	NECESSITY partner	1-3
Christophe Richez	Europe	Hôpital Pellegrin, CHU, Bordeaux, France	Clinician (rheumatology)	External	1
Alain Saraux	Europe	Hôpital de la Cavale Blanche, Brest, France	Clinician (rheumatology)	NECESSITY partner	1
**Raphaèle Seror**	Europe	**Assistance Publique Hôpitaux de Paris, France**	**Clinician (rheumatology)**	**NECESSITY partner, lead team**	**1-3**
Caroline Shiboski	America	University of California, San Francisco, USA	Clinician (dentistry)	External	1
Roser Solans Laque	Europe	Vall d'Hebron University Hospital, Barcelona, Spain	Clinician (rheumatology)	External	1
Muthiah Srinivasan	Asia	Aravind Eye Hospital, Madurai, Tamil Nadu, India	Clinician (ophthalmology)	External	1
Tsutomu Takeuchi	Asia	Keio University School of Medicine: Shinjuku-ku, Tokyo, Japan	Clinician (rheumatology)	External	1
Athanasios Tzioufas	Europe	School of Medicine, University of Athens, Greece	Clinician (rheumatology)	NECESSITY partner	1-3
Joel van Roon	Europe	UMC Utrecht, Utrecht University, Netherlands	Scientist	NECESSITY partner	1-3
Gwenny Verstappen	Europe	Universitair Medisch Centrum Groningen, Netherland	Scientist	NECESSITY partner	3
Arjan Vissink	Europe	Universitair Medisch Centrum Groningen, Netherland	Clinician (rheumatology, dentistry)	NECESSITY partner	1
Frederick Vivino	America	Penn Medicines, USA	Clinician (rheumatology)	External	1
Paraskevi Voulgari	Europe	University of Ioannina, Greece	Clinician (rheumatology)	External	1

Marie Wahren-Herlenius	Europe	Karolinska Institute, Sweden	Scientist	NECESSITY partner	1-3
Daniel Wallace	America	University of California Los Angeles and Cedars-Sinai Medical Center, USA	Clinician (rheumatology)	External	1
Ava Wu	America	University of California, San Francisco, USA	Clinician (oral medicine)	External	1
Wen Zhang	Asia	Peking Union Medical College Hospital, Beijing, China	Clinician (rheumatology)	External	1

Bold: lead team members

Patients:

The patients participated anonymously to the development of STAR.

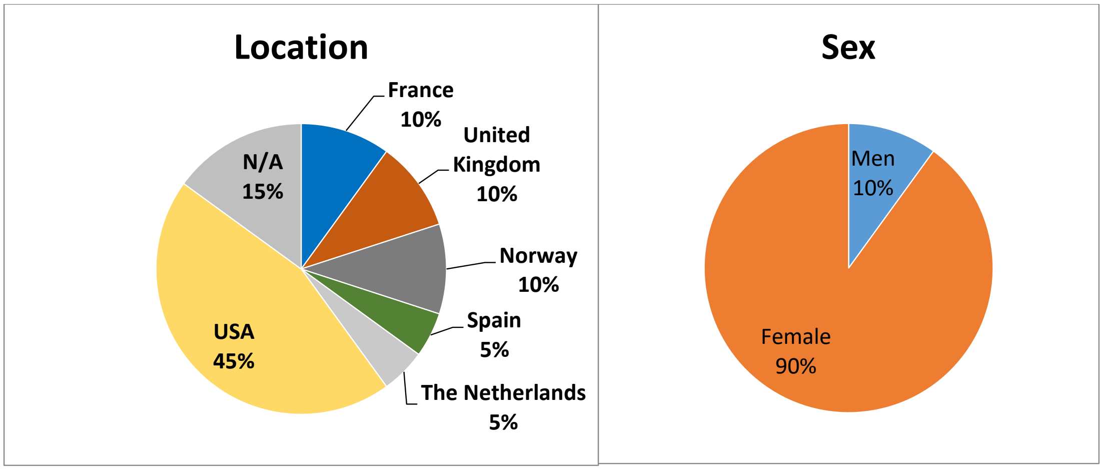

STEP 1: DEFINITION OF CORE SET

Supplement 2: Identification of responder subsets (Step 1): Baseline characteristics (variables) included in the virtual twins analysis for identifying subsets

	**Baseline characteristics**	
	**Set 1**	**Set 2**
Demographic data	Age, sex	Age, sex
Criteria suggested to better capture and objectify clinical improvement to treatment in previous trials	• Anti-SSA seropositivity<br>• Positive minor salivary gland biopsy (MSGB)<br>• Disease duration<br>• Dryness (salivary flow and Schirmer's test)<br>• B-cell hyperactivity, autoantibody titer and complement (biomarkers : IgG, Rheumatoid Factor (RF) (yes/no), low complement C4 (yes/no)),<br>• Lymphocyte count<br>• Erythrocyte Sedimentation Rate (ESR)	• Anti-SSA seropositivity<br>• Positive minor salivary gland biopsy (MSGB)<br>• Disease duration<br>• Dryness (salivary flow and Schirmer's test)<br>• B-cell hyperactivity, autoantibody titer and complement (biomarkers : IgG, Rheumatoid Factor (RF) (yes/no), low complement C4 (yes/no)),<br>• Lymphocyte count<br>• Erythrocyte Sedimentation Rate (ESR)
Scores	• Physician Global Assessment (PhGA) and Patient Global Assessment (PatGA)<br>• Oral and ocular dryness separately<br>• ESSDAI and ESSPRI total scores	• Physician Global Assessment (PhGA) and Patient Global Assessment (PatGA)<br>• Oral and ocular dryness separately<br>• ESSDAI and ESSPRI subscales (VAS dryness, pain, fatigue, ESSDAI domains)

Supplement 3: Identification of responder subsets (Step 1): Definitions of a response to treatment used in the virtual twins analysis for identifying subsets

Definition	Criteria
1	Improvement of ESSDAI ≥ 3
2	Improvement of ESSDAI ≥ 5
3	Improvement of ESSPRI ≥ 1
4	Improvement of ESSPRI ≥ 2
5	Improvement of ESSPRI ≥ 3
6	Improvement of PhGA ≥ 2
7	Improvement of PhGA ≥ 1
8	Improvement of PatGA ≥ 2
9	Improvement of PatGA ≥ 1
10	Improvement of PhGA or PatGA ≥ 2
11	Improvement of PhGA or PatGA ≥ 1
12	Any improvement of UWSF
13	Improvement of PhGA or PatGA ≥ 2 or any improvement of UWSF
14	Improvement of PhGA or PatGA ≥ 2 and any improvement of UWSF

ESSDAI: EULAR Sjögren syndrome disease activity index, ESSPRI: EULAR Sjögren syndrome patient reported index, PatGA: Patient Global Assessment, PhGA: Physician Global Assessment, UWSF: Unstimulated Whole Salivary Flow

Seror R, *et al. Ann Rheum Dis* 2022; 81:979–989. doi: 10.1136/annrheumdis-2021-222054

Supplement 4: Identified responder subsets and their characteristics (Step 1)

Responder subset	Definition of response	Baseline variables
Subset 1	Change in PhGA or PatGA ≥ 2	Set 1
Subset 2	Change in PhGA or PatGA ≥ 2 and any improvement of UWSF	Set 1
Subset 3	Change in PhGA or PatGA ≥ 2	Set 2
Subset 4	Change in PhGA or PatGA ≥ 2 or any improvement of UWSF	Set 2

PatGA: Patient Global Assessment, PhGA: Physician Global Assessment, UWSF: Unstimulated Whole Salivary Flow

	Combined rituximab trial population (N=253)	Subset 1 (N=125)	Subset 2 (N=91)	Subset 3 (N=100)	Subset 4 (N=83)
Age (years), median [IQR]	55.0 [45.0-63.0]	55.0 [46.0-63.0]	52.0 [45.0-61.0]	55.0 [45.0-64.5]	56.0 [47.0-67.0]
Women, n (%)	236 (93.3%)	119 (95.2%)	86 (94.5%)	94 (94.0%)	78 (94.0%)
Time since first symptoms (years), median [IQR]	7.0 [4.0-13.0]	7.1 [4.0-12.8]	5.9 [3.0-12.3]	9.0 [4.8-15.0]	8.3 [3.4-16.0]
Time since date of diagnosis (years), median [IQR]	4.0 [1.0-7.3]	3.3 [1.0-7.0]	3.0 [1.0-6.0]	3.7 [1.0-8.0]	3.0 [1.0-8.0]
Anti-Ro/SSA, n (%)	228 (90.1%)	113 (90.4%)	85 (93.4%)	93 (93.0%)	75 (90.4%)
Anti-La/SSB, n (%)	153 (61.4%)	86 (69.4%)	63 (70.8%)	62 (63.3%)	60 (73.2%)
Dryness (overall) (0-100), median [IQR]	76.0 [62.0-86.0]	78.0 [65.0-89.0]	71.0 [53.0-81.0]	86.5 [81.0-93.5]	77.0 [62.0-89.0]
Fatigue (0-100), median [IQR]	73.0 [61.0-84.0]	73.0 [60.0-83.0]	69.0 [60.0-81.0]	76.0 [64.0-87.5]	70.0 [60.0-80.0]
Joint pain (0-100), median [IQR]	58.0 [35.0-78.0]	53.0 [24.0-72.0]	55.0 [28.0-71.0]	57.0 [31.0-78.5]	53.0 [26.0-71.0]
ESSPRI (0-100), median [IQR]	67.3 [55.0-77.3]	65.0 [52.3-75.3]	62.0 [51.3-73.7]	72.0 [59.7-83.7]	64.7 [55.3-72.3]
Patient global activity (0-100), median [IQR]	72.0 [60.0-81.0]	71.0 [61.0-80.0]	68.0 [54.0-77.0]	76.5 [67.0-87.0]	70.0 [56.0-81.0]
ESSDAI score (0-123), mean (SD)	7.4 (6.3)	7.7 (6.2)	8.1 (6.6)	7.9 (7.0)	8.7 (7.1)
Physician global assessment (0-100), median [IQR]	50.0 [30.0-60.0]	60.0 [30.0-69.0]	50.0 [30.0-61.0]	50.0 [30.0-60.0]	50.0 [30.0-64.0]
Unstimulated salivary flow rate (ml/min), median [IQR]	0.1 [0.0-0.1]	0.1 [0.0-0.1]	0.1 [0.1-0.2]	0.0 [0.0-0.1]	0.0 [0.0-0.1]
Schirmer test (right eye) (mm), median [IQR]	5.0 [2.0-14.0]	5.0 [2.0-11.0]	7.0 [2.0-15.0]	4.0 [1.0-10.0]	5.0 [2.0-15.0]
Schirmer test (left eye) (mm), median [IQR]	5.0 [1.0-12.0]	5.0 [1.0-10.0]	6.5 [2.0-12.5]	4.0 [0.0-10.0]	5.0 [1.5-12.5]
Erythrocyte Sedimentation Rate (mm/hr), median [IQR]	22.0 [10.0-40.0]	25.0 [13.0-45.0]	35.0 [23.0-54.0]	32.0 [13.0-65.5]	48.0 [31.0-70.0]
Immunoglobulin G results(g/l), median [IQR]	15.9 [12.2-20.8]	17.5 [13.6-23.7]	18.0 [14.7-23.9]	17.9 [12.8-24.7]	19.3 [13.7-26.2]
Low C4, n (%)	22 (9.2%)	17 (4.4%)	7 (8.1%)	9 (9.5%)	7 (8.8%)
Lymphocytes (/mm3), median [IQR]	1344.9 (551.5)	1322.4 (511.3)	1253.5 (471.3)	1409.0 (591.0)	1368.2 (578.4)

ESSDAI: EULAR Sjögren syndrome disease activity index, ESSPRI: EULAR Sjögren syndrome patient reported index, IQR: interquartile range

Supplement 5: Identification of items sensitive to change (step 1): Effect size for the standardized difference in mean values at W24 of each outcome in the 4 responder subsets and the whole population of the two rituximab trials.

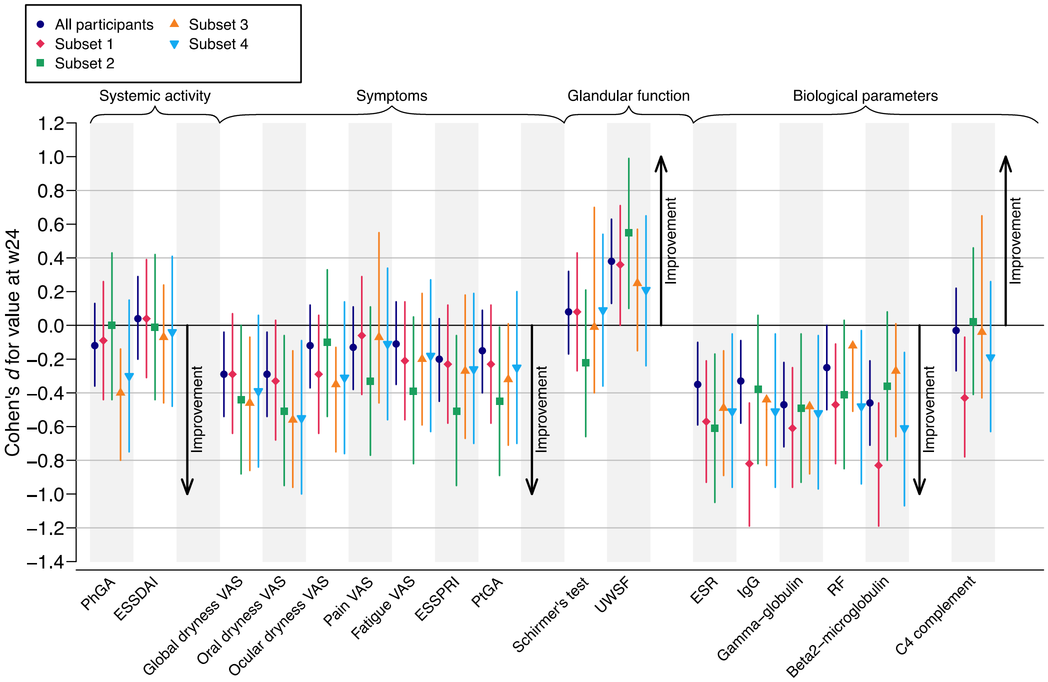

We performed a combined analysis of data from TEARS and TRACTISS rituximab trials. Cohen's Effect size and 95% CI for the standardized difference in mean values at W24 were computed for each outcome in the 4 responder subsets and in the whole population of the two rituximab trials.

ESR: Erythrocyte Sedimentation Rate; ESSDAI: EULAR Sjögren syndrome disease activity index, ESSPRI: EULAR Sjögren syndrome patient reported index, PtGA: Patient Global Assessment, PhGA: Physician Global Assessment, RF: Rheumatoid Factor, UWSF: Unstimulated Whole Salivary Flow

Supplement 6: Definition of core set of outcome measures: Experts and patients' scoring of items/domains (Step 1)

Delphi panellists were asked to rate the importance of measurement of each item in the context of assessing treatment response in clinical trials in pSS based on a 1 to 9 Likert scale, rated as follows: 1-3 not important, 4-6 important but not critical, 7-9 critical) and to provide comments, suggest new domains or measurements.

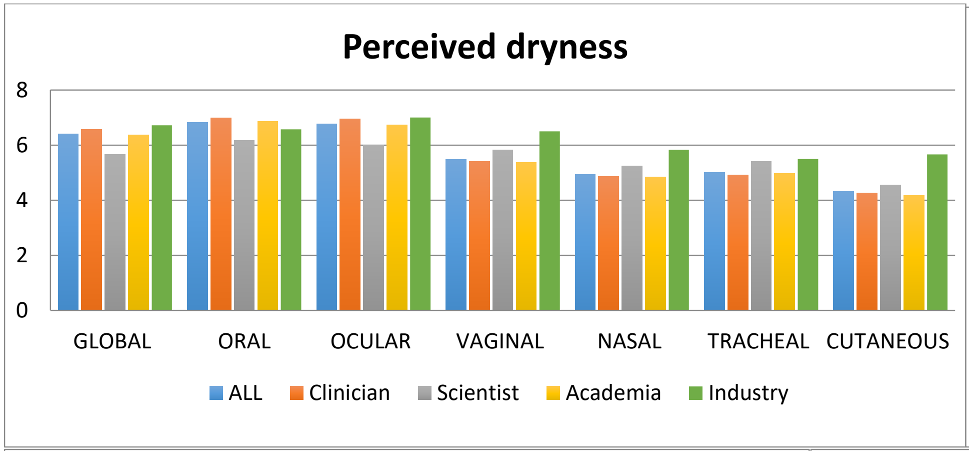

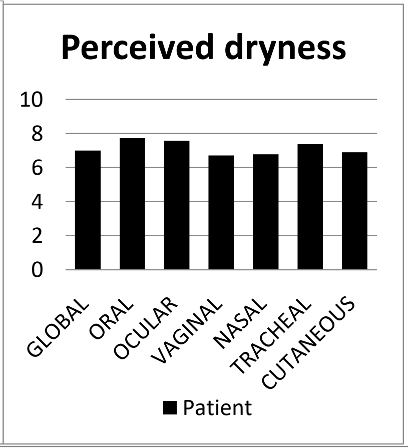

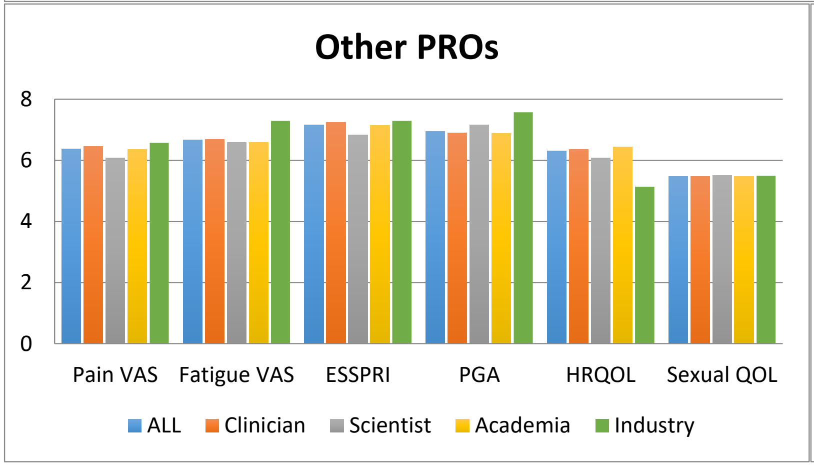

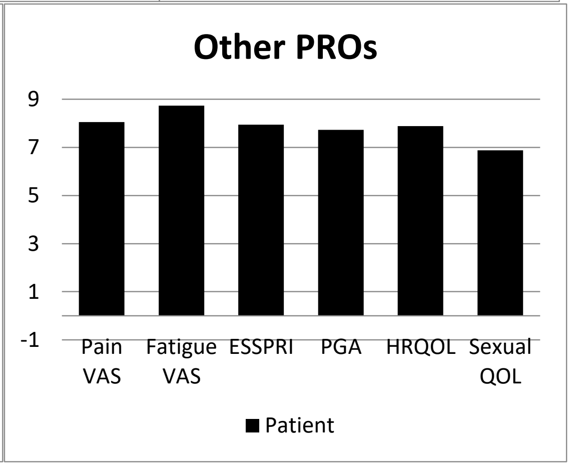

Seror R, *et al. Ann Rheum Dis* 2022; 81:979–989. doi: 10.1136/annrheumdis-2021-222054

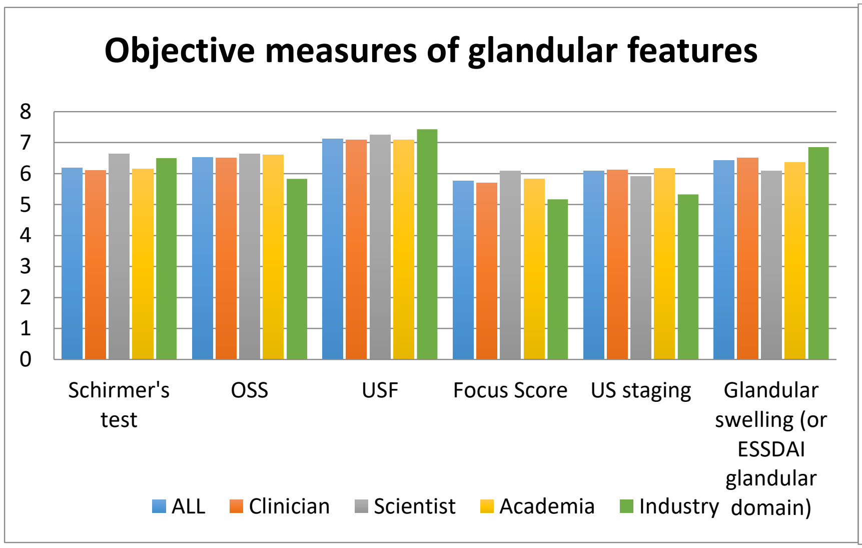
Objective measures of glandular features
8
7
6
5
4
3
2
1
0
Schirmer's test
OSS
USF
Focus Score
US staging
Glandular swelling (or ESSDAI glandular domain)
ALL
Clinician
Scientist
Academia
Industry

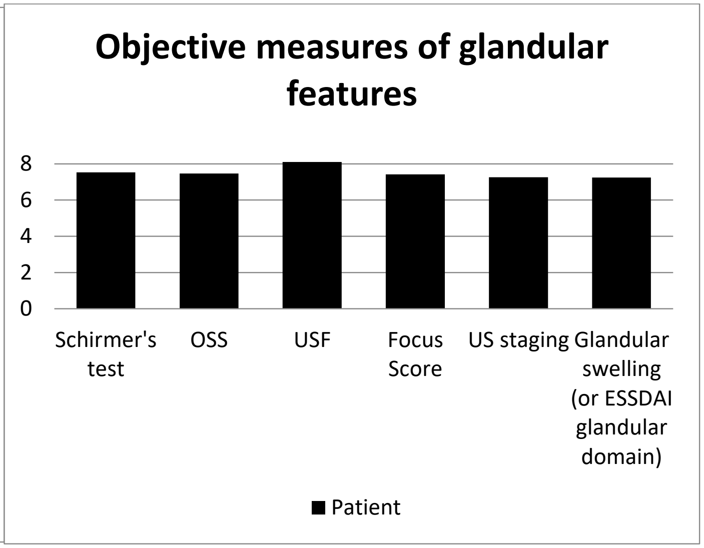
Objective measures of glandular features
8
6
4
2
0
Schirmer's test
OSS
USF
Focus Score
US staging
Glandular swelling (or ESSDAI glandular domain)
Patient

13

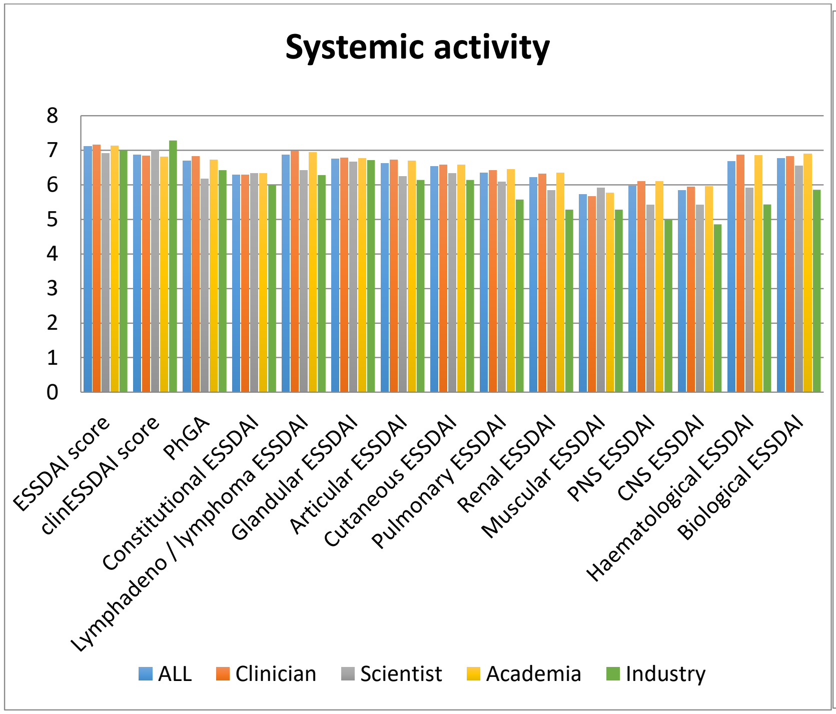
Systemic activity
8
7
6
5
4
3
2
1
0
ESSDAI score
clinESSDAI score
PhGA
Constitutional ESSDAI
Lymphadeno / lymphoma ESSDAI
Glandular ESSDAI
Articular ESSDAI
Cutaneous ESSDAI
Pulmonary ESSDAI
Renal ESSDAI
Muscular ESSDAI
PNS ESSDAI
CNS ESSDAI
Haematological ESSDAI
Biological ESSDAI
ALL
Clinician
Scientist
Academia
Industry

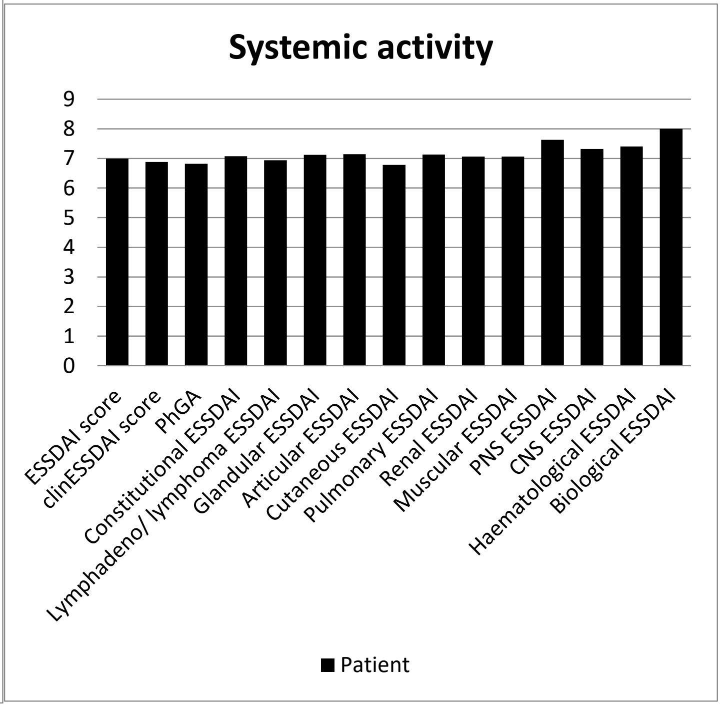
Systemic activity
9
8
7
6
5
4
3
2
1
0
ESSDAI score
clinESSDAI score
PhGA
Constitutional ESSDAI
Lymphadeno/ lymphoma ESSDAI
Glandular ESSDAI
Articular ESSDAI
Cutaneous ESSDAI
Pulmonary ESSDAI
Renal ESSDAI
Muscular ESSDAI
PNS ESSDAI
CNS ESSDAI
Haematological ESSDAI
Biological ESSDAI
Patient

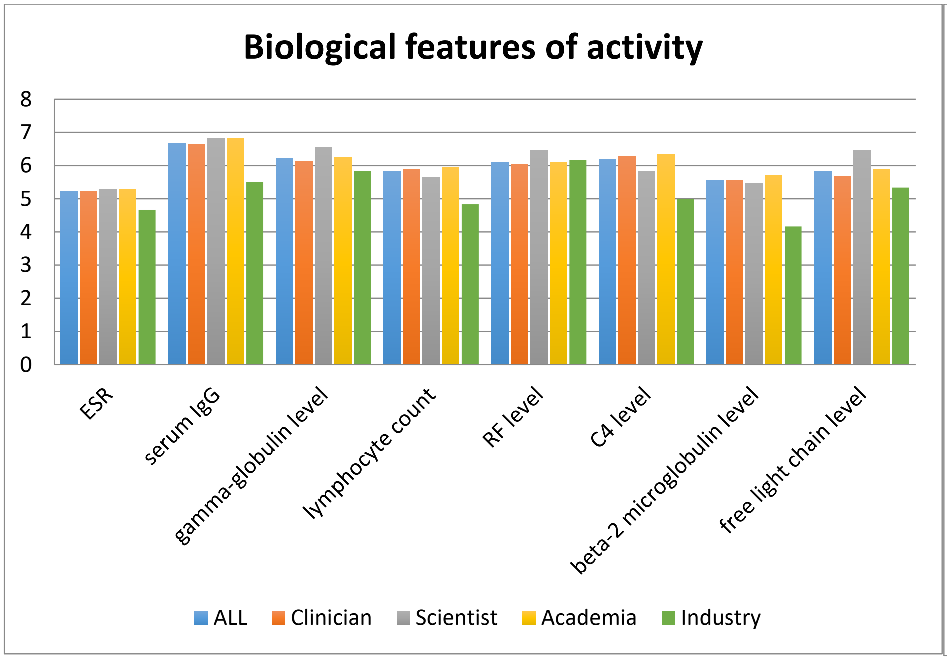
Biological features of activity
8
7
6
5
4
3
2
1
0
ESR
serum IgG
gamma-globulin level
lymphocyte count
RF level
C4 level
beta-2 microglobulin level
free light chain level
ALL
Clinician
Scientist
Academia
Industry

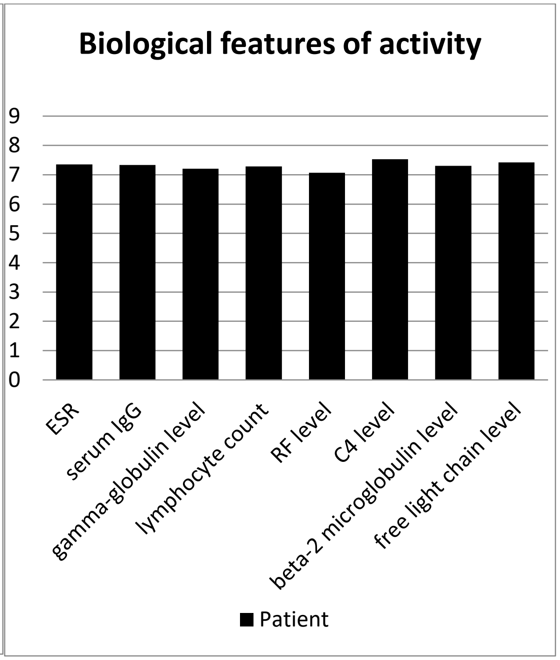
Biological features of activity
9
8
7
6
5
4
3
2
1
0
ESR
serum IgG
gamma-globulin level
lymphocyte count
RF level
C4 level
beta-2 microglobulin level
free light chain level
Patient

Supplement 7: Definition of core set of outcome measures (step 1): Results of patients and experts' vote and final selection

		Score ≥ 7 (% of participant)			Included in Core set (Y/N)	Rationale for inclusion
**Domain**	**Measurement instrument**	**Full panel**	**Experts**	**Patients**		
Disease activity	PhGA	55.3	56.9	50.0	N	
	ESSDAI	62.4	64.4	55.0	N	
	clinESSDAI score	54.1	55.4	50.0	Y	STAR includes a biological domain, so ESSDAI could not be used to avoid a redundant measurement of biological parameters. ClinESSDAI, which was the second most voted option, was selected. clinESSDAI is the ESSDAI score without the biological domain [Seror, 2016].
	ESSDAI-constitutional	45.9	46.2	45.0	N	
	ESSDAI-lymphadenopathy	57.6	58.5	55.0	N	
	ESSDAI-glandular	50.6	50.8	50.0	N	
	ESSDAI-articular	51.8	52.3	50.0	N	
	ESSDAI-cutaneous	43.5	43.1	45.0	N	
	ESSDAI-pulmonary	47.1	44.6	55.0	N	
	ESSDAI-renal	45.9	43.1	55.0	N	
	ESSDAI-muscular	40	35.4	55.0	N	
	ESSDAI-PNS	44.7	40.0	60.0	N	
	ESSDAI-CNS	42.4	38.5	55.0	N	
	ESSDAI-haematological	54.1	52.3	60.0	N	
	ESSDAI-biological	51.8	50.8	55.0	N	
Symptoms	Dryness-global	52.9	47.7	70.0	N	
	Dryness-oral	64.7	58.5	85.0	N	
	Dryness-ocular	62.4	56.9	80.0	N	
	Dryness-vaginal	30.6	23.1	55.0	N	
	Dryness-nasal	23.5	12.3	60.0	N	

Seror R, *et al. Ann Rheum Dis* 2022; 81:979–989. doi: 10.1136/annrheumdis-2021-222054

		Score ≥ 7 (% of participant)			Included in Core set (Y/N)	Rationale for inclusion
**Domain**	**Measurement instrument**	**Full panel**	**Experts**	**Patients**		
	Dryness-tracheal	29.4	16.9	70.0	N	
	Dryness-cutaneous	21.2	7.7	65.0	N	
	Pain	58.8	52.3	80.0	N	
	Fatigue	64.7	55.4	95.0	N	
	ESSPRI	75.3	73.8	80.0	Y	Individual oral and ocular dryness (along with pain and fatigue) were also included to be tested for the next step since they are relevant to patients and have a high sensitivity to change in some responder subsets
	PatGA	70.6	69.2	75.0	N	
	Health-related QOL	56.5	49.2	80.0	N	
	Sexual QOL	27.1	20.0	50.0	N	
Lachrymal gland function	Schirmer's test	42.4	36.9	60.0	Y	OSS is not widely available in clinical centers and can only be performed by trained ophthalmologists. Moreover it is an expensive technique that would significantly increase cost of studies. It was decided to also include the option of using the more widely available Schirmer's test, in the ocular domain along with the OSS, to increase the feasibility in all trials. This is in line with the OMERACT recommendation of feasibility.
	OSS	51.8	50.8	55.0	Y	
Salivary gland function	UWSF	71.8	67.7	85.0	Y	The experts decided to include US as an option. Although it is not currently feasible due to low availability and high requirement of expertise, the experts estimated that there was a significant benefit of this technique and that it could be expected to become more widely available in the future.
	Ultrasound	38.8	35.4	50.0	Y	
	Focus Score	35.3	29.2	55.0	N	
	Glandular swelling	54.1	52.3	60.0	N	

		Score ≥ 7 (% of participant)			Included in Core set (Y/N)	Rationale for inclusion
**Domain**	**Measurement instrument**	**Full panel**	**Experts**	**Patients**		
Biological parameters	ESR	23.5	15.4	50.0	N	
	Lymphocyte count	31.8	24.6	55.0	N	
	Serum IgG levels	56.5	56.9	55.0	Y	Both IgG and RF levels were included as biological markers of activity since they were clinically relevant, sensitive to change and routinely available. Although C4 complement had a higher score than RF, it was decided not to include it because it is quite redundant with RF but less feasible.
	Rheumatoid Factor levels	44.7	41.5	55.0	Y	
	Gamma-globulin	43.5	40.0	55.0	N	
	C4 complement	47.1	43.1	60.0	N	
	Beta-2 microglobulin	25.9	20.0	45.0	N	
	Free light chain	29.4	23.1	50.0	N	

ESSDAI: EULAR Sjögren syndrome disease activity index, ESSPRI: EULAR Sjögren syndrome patient reported index, OSS: Ocular Staining Score, PatGA: Patient Global Assessment, PhGA: Physician Global Assessment, RF: Rheumatoid Factor,US: Ultrasound, UWSF: Unstimulated Whole Salivary Flow

Seror R, *et al. Ann Rheum Dis* 2022; 81:979–989. doi: 10.1136/annrheumdis-2021-222054

STEP 2:CONSTRUCTION OF STAR OPTIONS

Supplement 8: Construction of STAR options (step2): Description of options tested for sensitivity to change

<table>
<tr><th>Proposal</th><th>Description</th><th>Variations to be tested</th></tr>
<tr><td>STAR design 1 (DAS-like)</td><td>α * clinESSDAI + β * ESSPRI + χ* UWSF + δ * Schirmer + ϕ * IgG
Either PhGA or PatGA used as gold standard.</td><td></td></tr>
<tr><td>STAR design 2 (SRI-like)</td><td>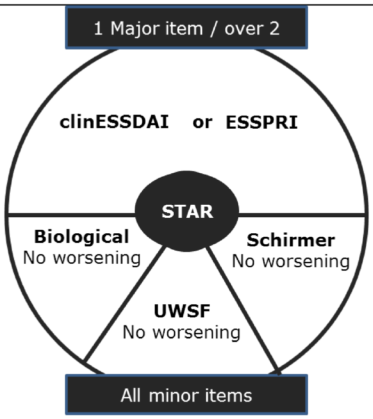

Responder =
improvement in 1 major and no worsening of the others</td><td>- Δ clinESSDAI ≥ 3
- Δ clinESSDAI ≥ 5
- Improvement ESSPRI ≥ 1 point or 15%
- Improvement ESSPRI ≥ 20 %
- Improvement of 15% of 2/3 VAS (dryness, pain, fatigue)
- Improvement of 30% of 2/3 VAS (dryness, pain, fatigue)
- Improvement of 15% of 2/4 VAS (ocular dryness, oral dryness, pain, fatigue)
- Improvement of 30% of 2/3 VAS (ocular dryness, oral dryness, pain, fatigue)
- No worsening IgG
- No worsening IgG and RF</td></tr>
<tr><td>STAR design 3A</td><td><table>
<tr><th colspan="2">Domain</th><th>Measure</th><th>Definition of response</th></tr>
<tr><td>1. Systemic activity</td><td>MAJOR</td><td>ClinESSDAI</td><td></td></tr>
<tr><td>2. Patient reported outcome</td><td>MAJOR</td><td>ESSPRI</td><td></td></tr>
<tr><td>3. Lachrymal gland function</td><td>MINOR</td><td>Schirmer</td><td>If abnormal score at baseline, increase of ≥ 5 mm
If normal score at baseline, no change to abnormal</td></tr>
<tr><td>4. Salivary gland function</td><td>MINOR</td><td>UWSF</td><td>Increase of ≥25%, or if score is 0 at baseline any increase in UWSF</td></tr>
<tr><td>5. Biological</td><td>MINOR</td><td>IgG</td><td></td></tr>
</table>Responder: ≥ 3 domains with at least 1 major domain</td><td>- Δ clinESSDAI ≥ 3
- Δ clinESSDAI ≥ 5
- Improvement ESSPRI ≥ 1 point or 15%
- Improvement ESSPRI ≥ 20 %
- Improvement of 15% of 2/3 VAS (dryness, pain, fatigue)
- Improvement of 30% of 2/3 VAS (dryness, pain, fatigue)
- Improvement of 15% of 2/4 VAS (ocular dryness, oral dryness, pain, fatigue)
- Improvement of 30% of 2/3 VAS (ocular dryness, oral dryness, pain, fatigue)
- IgG responder (decrease of ≥ 10 %)
- No worsening IgG</td></tr>
</table>

<table>
<tr>
<td>STAR design 3B</td>
<td>
<table>
<tr><th>Domain</th><th></th><th>Measure</th><th>Definition of response</th></tr>
<tr><td>1. Systemic activity</td><td>MAJOR (3 points)</td><td>ClinESSDAI</td><td></td></tr>
<tr><td>2. Patient reported outcome</td><td>MAJOR (3 points)</td><td>ESSPRI</td><td></td></tr>
<tr><td>3. Lachrymal gland function</td><td>MINOR (1 point)</td><td>Schirmer</td><td>If abnormal score at baseline, increase of ≥ 5 mm
If normal score at baseline, no change to abnormal</td></tr>
<tr><td>4. Salivary gland function</td><td>MINOR (1 point)</td><td>UWSF</td><td>Increase of ≥25%, or if score is 0 at baseline any increase in UWSF</td></tr>
<tr><td>5. Biological</td><td>MINOR (1 point)</td><td>IgG</td><td></td></tr>
</table>
</td>
<td>
- Δ clinESSDAI ≥ 3
- Δ clinESSDAI ≥ 5
- Improvement ESSPRI ≥ 1 point or 15%
- Improvement ESSPRI ≥ 20 %
- Improvement of 15% of 2/3 VAS (dryness, pain, fatigue)
- Improvement of 30% of 2/3 VAS (dryness, pain, fatigue)
- Improvement of 15% of 2/4 VAS (ocular dryness, oral dryness, pain, fatigue)
- Improvement of 30% of 2/3 VAS (ocular dryness, oral dryness, pain, fatigue)
- IgG responder (decrease of ≥ 10 %)
- No worsening IgG (worsening defined as increase of ≥ 10% IgG level. If IgG=0 at baseline, then any increase is a worsening)
- Scoring options: 3 points for major domain; 1 point for minor domain
  - Binary score: test responder ≥ 3, 4, 5, 6, or 7 points
  - Continuous : ordinal scale
</td>
</tr>
<tr>
<td>STAR design 4 (ACR-like)</td>
<td>
<table>
<tr><th>Domain</th><th></th><th>Measurement</th></tr>
<tr><td>1. Systemic activity</td><td>MAJOR</td><td>ClinESSDAI</td></tr>
<tr><td>2. Patient reported outcome</td><td>MAJOR</td><td>ESSPRI</td></tr>
<tr><td>3. Lachrymal gland function</td><td>MINOR</td><td>Schirmer</td></tr>
<tr><td>4. Salivary gland function</td><td>MINOR</td><td>UWSF</td></tr>
<tr><td>5. Biological</td><td>MINOR</td><td>IgG</td></tr>
</table>
Responder: % of improvement in X of 5 domains with at least 1 major
</td>
<td>
- Improvement in 2 of 5 domains
- Improvement in 3 of 5 domains
- 10% of improvement
- 20% of improvement
- 30% of improvement
- 40% of improvement
- 50% of improvement
- 60% of improvement
- 70% of improvement
- 10% of improvement for ESSPRI and IgG, 20% for the other domains
- 10% of improvement for ESSPRI and IgG, 30% for the other domains
- No worsening of the other major domain
</td>
</tr>
</table>

CRESS

Item	Measurement	Definition of response
Systemic disease activity	ClinESSDAI	Score < 5 (low disease activity)
Patient-reported symptoms	ESSPRI	Decrease of ≥1 point or ≥15% from baseline
Tear gland	OSS/Schirmer	If abnormal score baseline:<br>• Decrease of ≥ 2 points in OSS<br>• Or increase of ≥ 5 mm in Schirmer<br>Or if both normal score baseline<br>• No change to abnormal in OSS and Schirmer
Salivary gland	UWS/US	• Increase of ≥ 25%, or if score is 0 at baseline any increase in UWS<br>• Or decrease of ≥ 25% in total Hocevar score (US)
Serological	RF/IgG	• Decrease of ≥ 25% in RF<br>• Or decrease of ≥ 10% in IgG
**CRESS responder**		**≥ 3 of 5 items responder**

Follow-up visit was week 24 for all trials, except ETAP and anti-CD40 (week 12) and TRACTISS (week 48).

In the sensitivity to change analysis, only Schirmer's was used for the scoring of lachrymal gland function domain because OSS was not available in most of the trials. Results of Schirmer's test were not available for the anti-BAFFR trial so OSS score was used instead.

Only UWSF was used for the scoring of salivary gland function because Ultrasound data were not available in most of the trials.

Only IgG levels was used for the scoring of the biological domain because the dosage and results of RF levels were not available or not homogeneously recorded in all trials.

The concise CRESS (CRESS without ultrasound and OSS measurements) was used for analysis.

ESSDAI: EULAR Sjögren syndrome disease activity index, ESSPRI: EULAR Sjögren syndrome patient reported index, OSS: Ocular Staining Score, PatGA: Patient Global Assessment, PhGA: Physician Global Assessment, RF: Rheumatoid Factor, US: Ultrasound, UWSF: Unstimulated Whole Salivary Flow, VAS: visual Analog Scale

STEP 3: EVALUATION OF SENSITIVITY TO CHANGE OF STAR OPTIONS AND SELECTION OF THE CANDIDATE STAR

Supplement 9: Sensitivity to change for STAR options: Example of results of analysis of sensitivity to change for STAR design 3B binary score threshold 5 and 4

Version	Systemic activity	Patient Reported Outcome	Ocular gland function	Oral gland function	Biological
V1	ΔclinESSDAI≥3	ESSPRI improvement ≥15%	Schirmer responder	UWSF responder	IgG responder
V2	ΔclinESSDAI≥3	ESSPRI improvement ≥20%	Schirmer responder	UWSF responder	IgG responder
V3	ΔclinESSDAI≥3	15% improvement of 2 of 3 VAS	Schirmer responder	UWSF responder	IgG responder
V4	ΔclinESSDAI≥3	30% improvement of 2 of 3 VAS	Schirmer responder	UWSF responder	IgG responder
V5	ΔclinESSDAI≥3	15% improvement of 2 of 4 VAS	Schirmer responder	UWSF responder	IgG responder
V6	ΔclinESSDAI≥3	30%improvement of 2 of 4 VAS	Schirmer responder	UWSF responder	IgG responder
V7	ΔclinESSDAI≥3	ESSPRI improvement ≥15%	Schirmer responder	UWSF responder	No worsening of IgG
V8	ΔclinESSDAI≥3	ESSPRI improvement ≥20 %	Schirmer responder	UWSF responder	No worsening of IgG
V9	ΔclinESSDAI≥3	15% improvement of 2 of 3 VAS	Schirmer responder	UWSF responder	No worsening of IgG
V10	ΔclinESSDAI≥3	30%improvement of 2 of 3 VAS	Schirmer responder	UWSF responder	No worsening of IgG
V11	ΔclinESSDAI≥3	15% improvement of 2 of 4 VAS	Schirmer responder	UWSF responder	No worsening of IgG
V12	ΔclinESSDAI≥3	30%improvement of 2 of 4 VAS	Schirmer responder	UWSF responder	No worsening of IgG
V13	ΔclinESSDAI≥5	ESSPRI improvement ≥15%	Schirmer responder	UWSF responder	IgG responder
V14	ΔclinESSDAI≥5	ESSPRI improvement ≥20%	Schirmer responder	UWSF responder	IgG responder
V15	ΔclinESSDAI≥5	15% improvement of 2 of 3 VAS	Schirmer responder	UWSF responder	IgG responder
V16	ΔclinESSDAI≥5	30%improvement of 2 of 3 VAS	Schirmer responder	UWSF responder	IgG responder
V17	ΔclinESSDAI≥5	15% improvement of 2 of 4 VAS	Schirmer responder	UWSF responder	IgG responder
V18	ΔclinESSDAI≥5	30%improvement of 2 of 4 VAS	Schirmer responder	UWSF responder	IgG responder
V19	ΔclinESSDAI≥5	ESSPRI improvement ≥15%	Schirmer responder	UWSF responder	No worsening of IgG

V20	ΔclinESSDAI≥5	ESSPRI improvement ≥ 20%	Schirmer responder	UWSF responder	No worsening of IgG
V21	ΔclinESSDAI≥5	15% improvement of 2 of 3 VAS	Schirmer responder	UWSF responder	No worsening of IgG
V22	ΔclinESSDAI≥5	30% improvement of 2 of 3 VAS	Schirmer responder	UWSF responder	No worsening of IgG
V23	ΔclinESSDAI≥5	15% improvement of 2 of 4 VAS	Schirmer responder	UWSF responder	No worsening of IgG
V24	ΔclinESSDAI≥5	30% improvement of 2 of 4 VAS	Schirmer responder	UWSF responder	No worsening of IgG

Seror R, *et al. Ann Rheum Dis* 2022; 81:979–989. doi: 10.1136/annrheumdis-2021-222054

Representation of responder patients (green) and non-responder patients (red) according to each of the 24 versions of STAR design 3B <u>threshold 5</u> in treatment and placebo arms in each RCT available for analysis.

V1 is the candidate STAR. White squares represent patients for whom the score could not be calculated because of missing data.

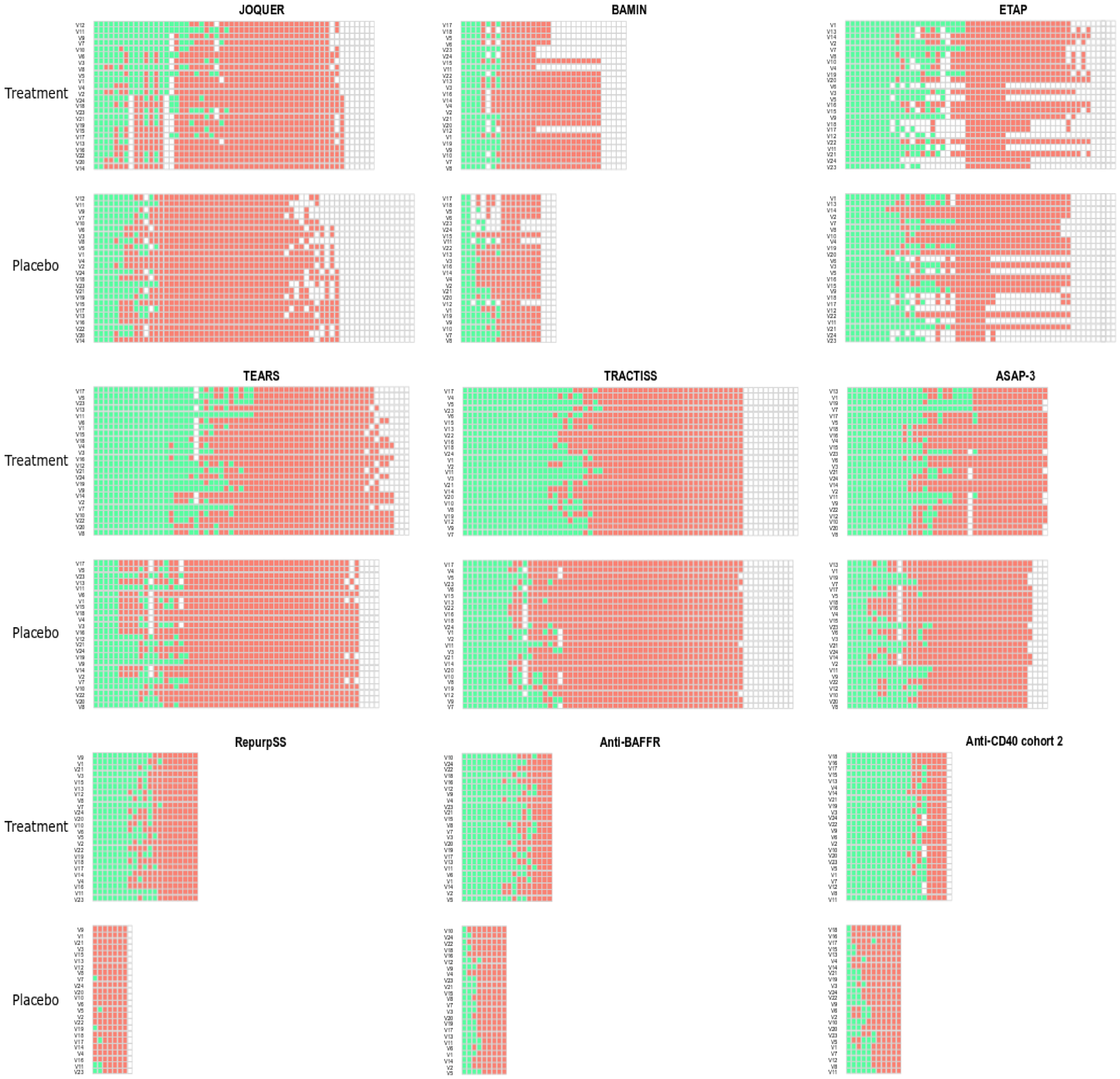

Representation of responder patients (green) and non-responder patients (red) according to each of the 24 versions of STAR design 3B threshold 4 in treatment and placebo arms in each RCT available for analysis.

White squares represent patients for whom the score could not be calculated because of missing data.

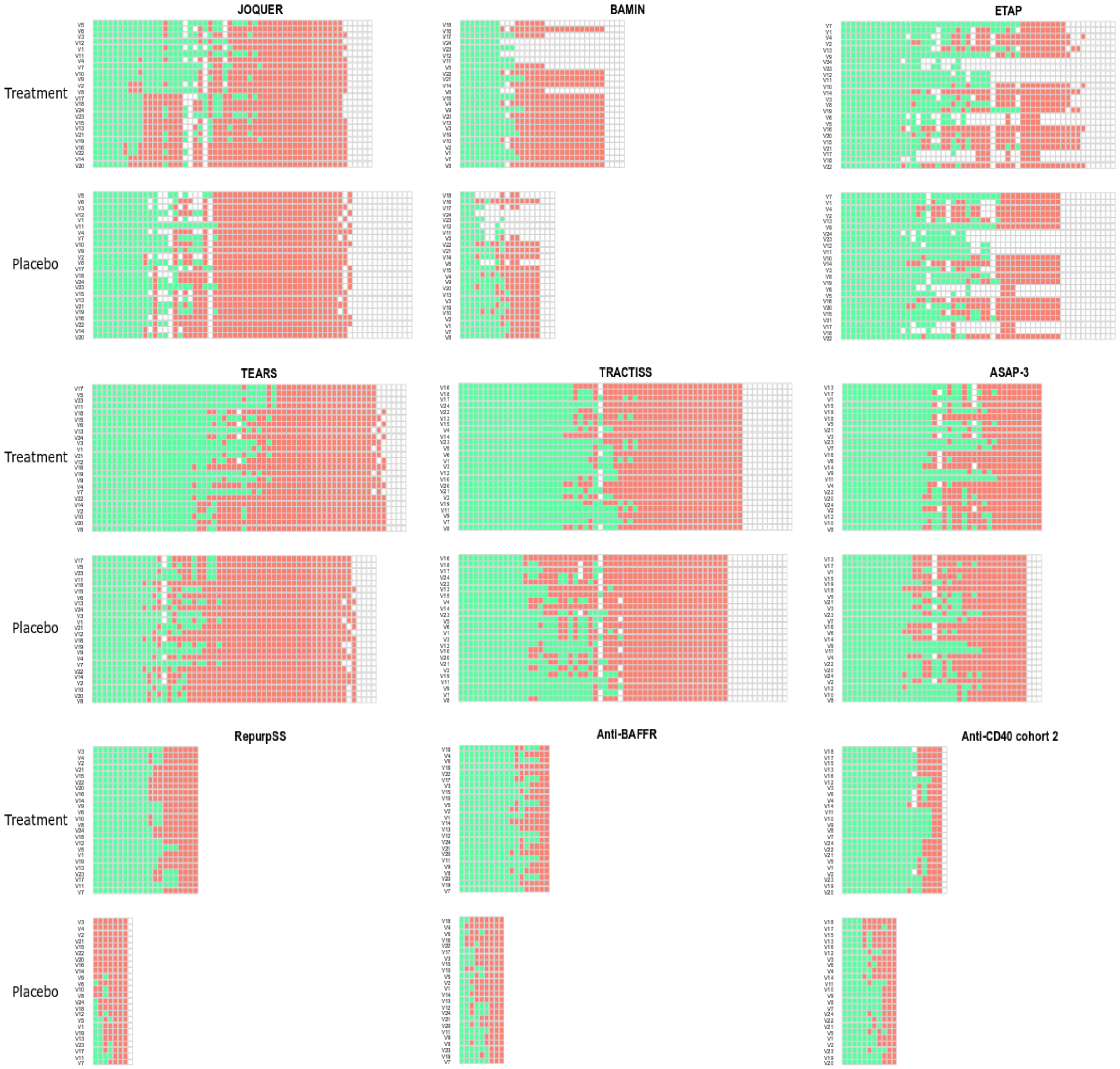

Seror R, *et al. Ann Rheum Dis* 2022; 81:979–989. doi: 10.1136/annrheumdis-2021-222054

Supplement 10: Classification of trials as ‘positive’ or ‘negative’: Results of vote

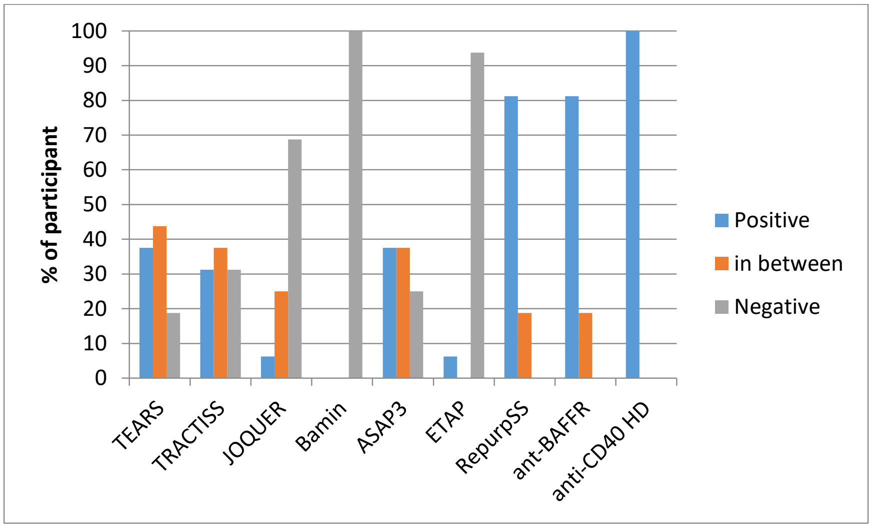

Supplement 11: Description of STAR alternate options

STAR design 3B threshold 5 V17

Domain	Point	Definition of response
**Systemic activity**	3	Decrease of clinESSDAI ≥ 5
**Patient reported outcome** *(assessed by VAS of ocular dryness, oral dryness, fatigue and pain)*	3	Decrease of 15% in at least 2 of 4 VAS
**Lachrymal gland function** *(assessed by Schirmer's test or Ocular Staining Score)*	1	Schirmer:<br>If abnormal score at baseline: increase ≥ 5 mm<br>If normal score at baseline: no change to abnormal<br>**or**<br>Ocular Staining Score:<br>If abnormal score at baseline: decrease ≥ 2 points<br>If normal score at baseline: no change to abnormal
**Salivary gland function** *(assessed by unstimulated whole salivary flow or ultrasound)*	1	Unstimulated Whole Salivary Flow:<br>If score > 0 at baseline: increase ≥ 25%<br>If score is 0 at baseline: any increase in UWSF<br>**or**<br>Ultrasound:<br>Decrease ≥ 25% in total Hocevar score
**Biological** *(assessed by IgG or RF)*	1	IgG: decrease ≥ 10%<br>**or**<br>Rheumatoid factor: decrease ≥ 25%
**Responder**		**≥ 5 points**

STAR design 3B threshold 5 V13

Domain	Point	Definition of response
**Systemic activity**	3	Decrease of clinESSDAI ≥ 5
**Patient reported outcome**	3	Decrease of ESSPRI ≥ 1 point or ≥ 15%
**Lachrymal gland function** *(assessed by Schirmer's test or Ocular Staining Score)*	1	Schirmer:<br>If abnormal score at baseline: increase ≥ 5 mm<br>If normal score at baseline: no change to abnormal<br>**or**<br>Ocular Staining Score:<br>If abnormal score at baseline: decrease ≥ 2 points<br>If normal score at baseline: no change to abnormal
**Salivary gland function** *(assessed by unstimulated whole salivary flow or ultrasound)*	1	Unstimulated Whole Salivary Flow:<br>If score > 0 at baseline: increase ≥ 25%<br>If score is 0 at baseline: any increase in UWSF<br>**or**<br>Ultrasound:<br>Decrease ≥ 25% in total Hocevar score

**Biological**<br>*(assessed by IgG or RF)*	1	IgG: decrease ≥ 10%<br>**or**<br>Rheumatoid factor: decrease ≥ 25%
**Responder**		**≥ 5 points**

STAR design 3A V1

Domain	Level	Definition of response
**Systemic activity**	Major	Decrease of clinESSDAI ≥ 5
**Patient reported outcome**	Major	Decrease of ESSPRI ≥ 1 point or ≥ 15%
**Lachrymal gland function**<br>*(assessed by Schirmer's test or Ocular Staining Score)*	Minor	Schirmer:<br>If abnormal score at baseline: increase ≥ 5 mm<br>If normal score at baseline: no change to abnormal<br>**or**<br>Ocular Staining Score:<br>If abnormal score at baseline: decrease ≥ 2 points<br>If normal score at baseline: no change to abnormal
**Salivary gland function**<br>*(assessed by unstimulated whole salivary flow or ultrasound)*	Minor	Unstimulated Whole Salivary Flow:<br>If score > 0 at baseline: increase ≥ 25%<br>If score is 0 at baseline: any increase in UWSF<br>**or**<br>Ultrasound:<br>Decrease ≥ 25% in total Hocevar score
**Biological**<br>*(assessed by IgG or RF)*	Minor	IgG: decrease ≥ 10%<br>**or**<br>Rheumatoid factor: decrease ≥ 25%
**Responder**		**≥ 3 of 5 domains including at least 1 major domain**

CRESS

Domain	Definition of response
**Systemic activity**	clinESSDAI < 5
**Patient reported outcome**	Decrease of ESSPRI ≥ 1 point or ≥ 15%
**Lachrymal gland function**<br>*(assessed by Schirmer's test or Ocular Staining Score)*	Schirmer:<br>If abnormal score at baseline:increase ≥ 5 mm<br>If normal score at baseline: no change to abnormal<br>**or**<br>Ocular Staining Score:<br>If abnormal score at baseline: decrease ≥ 2 points<br>If normal score at baseline: no change to abnormal

Seror R, *et al. Ann Rheum Dis* 2022; 81:979–989. doi: 10.1136/annrheumdis-2021-222054

**Salivary gland function**<br>*(assessed by unstimulated whole salivary flow or ultrasound)*	Unstimulated Whole Salivary Flow:<br>If score > 0 at baseline: increase ≥ 25%<br>If score is 0 at baseline: any increase in UWSF<br>**<u>or</u>**<br>Ultrasound:<br>Decrease ≥ 25% in total Hocevar score
**Biological**<br>*(assessed by IgG or RF)*	IgG: decrease ≥ 10%<br>**<u>or</u>**<br>Rheumatoid factor: decrease ≥ 25%
**CRESS responder**	**≥ 3 of 5 domains**

STAR design 3B threshold 5 V5

Domain	Point	Definition of response
**Systemic activity**	3	Decrease of clinESSDAI ≥ 3
**Patient reported outcome**<br>*(assessed by VAS of ocular dryness, oral dryness, fatigue and pain)*	3	Decrease of 15% in at least 2 of 4 VAS
**Lachrymal gland function**<br>*(assessed by Schirmer's test or Ocular Staining Score)*	1	Schirmer:<br>If abnormal score at baseline: increase ≥ 5 mm<br>If normal score at baseline: no change to abnormal<br>**<u>or</u>**<br>Ocular Staining Score:<br>If abnormal score at baseline: decrease ≥ 2 points<br>If normal score at baseline: no change to abnormal
**Salivary gland function** *(assessed by unstimulated whole salivary flow or ultrasound)*	1	Unstimulated Whole Salivary Flow:<br>If score > 0 at baseline: increase ≥ 25%<br>If score is 0 at baseline: any increase in UWSF<br>**<u>or</u>**<br>Ultrasound:<br>Decrease ≥ 25% in total Hocevar score
**Biological**<br>*(assessed by IgG or RF)*	1	IgG: decrease ≥ 10%<br>**<u>or</u>**<br>Rheumatoid factor: decrease ≥ 25%
	**Responder**	**≥ 5 points**

STAR design 3B continuous V1

Domain	Point	Definition of response
**Systemic activity**	3	Decrease of clinESSDAI ≥ 3
**Patient reported outcome**	3	Decrease of ESSPRI ≥ 1 point or ≥ 15%

**Lachrymal gland function**<br>*(assessed by Schirmer's test or Ocular Staining Score)*	1	Schirmer:<br>If abnormal score at baseline: increase ≥ 5 mm<br>If normal score at baseline: no change to abnormal<br>**or**<br>Ocular Staining Score:<br>If abnormal score at baseline: decrease ≥ 2 points<br>If normal score at baseline: no change to abnormal
**Salivary gland function**<br>*(assessed by unstimulated whole salivary flow or ultrasound)*	1	Unstimulated Whole Salivary Flow:<br>If score > 0 at baseline: increase ≥ 25%<br>If score is 0 at baseline: any increase in UWSF<br>**or**<br>Ultrasound:<br>Decrease ≥ 25% in total Hocevar score
**Biological**<br>*(assessed by IgG or RF)*	1	IgG: decrease ≥ 10%<br>**or**<br>Rheumatoid factor: decrease ≥ 25%
	**Score**	**Change from baseline between groups**

STAR design 3A V5

**Domain**	**Level**	**Definition of response**
**Systemic activity**	Major	Decrease of clinESSDAI ≥ 3
**Patient reported outcome**<br>*(assessed by VAS of ocular dryness, oral dryness, fatigue and pain)*	Major	Decrease of 15% in at least 2 of 4 VAS
**Lachrymal gland function**<br>*(assessed by Schirmer's test or Ocular Staining Score)*	Minor	Schirmer:<br>If abnormal score at baseline: increase ≥ 5 mm<br>If normal score at baseline: no change to abnormal<br>**or**<br>Ocular Staining Score:<br>If abnormal score at baseline: decrease ≥ 2 points<br>If normal score at baseline: no change to abnormal
**Salivary gland function**<br>*(assessed by unstimulated whole salivary flow or ultrasound)*	Minor	Unstimulated Whole Salivary Flow:<br>If score > 0 at baseline: increase ≥ 25%<br>If score is 0 at baseline: any increase in UWSF<br>**or**<br>Ultrasound:<br>Decrease ≥ 25% in total Hocevar score
**Biological**<br>*(assessed by IgG or RF)*	Minor	IgG: decrease ≥ 10%<br>**or**<br>Rheumatoid factor: decrease ≥ 25%
	**Responder**	**≥ 3 of 5 domains including at least 1 major domain**

Seror R, *et al. Ann Rheum Dis* 2022; 81:979–989. doi: 10.1136/annrheumdis-2021-222054

STAR design 4 V29

Domain	Definition of response
**Systemic activity**	Improvement of clinESSDAI ≥ 20%
**Patient reported outcome**	Improvement of ESSPRI ≥ 10%
**Lachrymal gland function** *(assessed by Schirmer's test or Ocular Staining Score)*	Improvement of Schirmer score ≥ 20%<br>**or**<br>Improvement of Ocular Staining Score ≥ 20%
**Salivary gland function** *(assessed by unstimulated whole salivary flow or ultrasound)*	Improvement of Unstimulated Whole Salivary Flow score ≥ 20%<br>**or**<br>Improvement of total Hocevar score ≥ 20%
**Biological** *(assessed by IgG or RF)*	Improvement in IgG level ≥ 10%<br>**or**<br>Improvement in Rheumatoid factor level ≥ 20%
**Responder**	**Improvement in ≥ 3 of 5 domains**

STAR design 3B threshold 6 V5

Domain	Point	Definition of response
**Systemic activity**	3	Decrease of clinESSDAI ≥ 3
**Patient reported outcome** *(assessed by VAS of ocular dryness, oral dryness, fatigue and pain)*	3	Decrease of 15% in at least 2 of 4 VAS
**Lachrymal gland function** *(assessed by Schirmer's test or Ocular Staining Score)*	1	Schirmer:<br>If abnormal score at baseline: increase ≥ 5 mm<br>If normal score at baseline: no change to abnormal<br>**or**<br>Ocular Staining Score:<br>If abnormal score at baseline: decrease ≥ 2 points<br>If normal score at baseline: no change to abnormal
**Salivary gland function** *(assessed by unstimulated whole salivary flow or ultrasound)*	1	Unstimulated Whole Salivary Flow:<br>If score > 0 at baseline: increase ≥ 25%<br>If score is 0 at baseline: any increase in UWSF<br>**or**<br>Ultrasound:<br>Decrease ≥ 25% in total Hocevar score
**Biological** *(assessed by IgG or RF)*	1	IgG: decrease ≥ 10%<br>**or**<br>Rheumatoid factor: decrease ≥ 25%
	**Responder**	**≥ 6 points**

Seror R, *et al. Ann Rheum Dis* 2022; 81:979–989. doi: 10.1136/annrheumdis-2021-222054

STAR design 3B threshold 6 V17

Domain	Point	Definition of response
**Systemic activity**	3	Decrease of clinESSDAI ≥ 5
**Patient reported outcome** *(assessed by VAS of ocular dryness, oral dryness, fatigue and pain)*	3	Decrease of 15% in at least 2 of 4 VAS
**Lachrymal gland function** *(assessed by Schirmer's test or Ocular Staining Score)*	1	Schirmer:<br>If abnormal score at baseline: increase ≥ 5 mm<br>If normal score at baseline: no change to abnormal<br>**or**<br>Ocular Staining Score:<br>If abnormal score at baseline: decrease ≥ 2 points<br>If normal score at baseline: no change to abnormal
**Salivary gland function** *(assessed by unstimulated whole salivary flow or ultrasound)*	1	Unstimulated Whole Salivary Flow:<br>If score > 0 at baseline: increase ≥ 25%<br>If score is 0 at baseline: any increase in UWSF<br>**or**<br>Ultrasound:<br>Decrease ≥ 25% in total Hocevar score
**Biological** *(assessed by IgG or RF)*	1	IgG: decrease ≥ 10%<br>**or**<br>Rheumatoid factor: decrease ≥ 25%
	**Responder**	**≥ 6 points**

STAR design 3B continuous V13

Domain	Point	Definition of response
**Systemic activity**	3	Decrease of clinESSDAI ≥ 5
**Patient reported outcome**	3	Decrease of ESSPRI ≥ 1 point or ≥ 15%
**Lachrymal gland function** *(assessed by Schirmer's test or Ocular Staining Score)*	1	Schirmer:<br>If abnormal score at baseline: increase ≥ 5 mm<br>If normal score at baseline: no change to abnormal<br>**or**<br>Ocular Staining Score:<br>If abnormal score at baseline: decrease ≥ 2 points<br>If normal score at baseline: no change to abnormal
**Salivary gland function** *(assessed by unstimulated whole salivary flow or ultrasound)*	1	Unstimulated Whole Salivary Flow:<br>If score > 0 at baseline: increase ≥ 25%<br>If score is 0 at baseline: any increase in UWSF<br>**or**<br>Ultrasound:<br>Decrease ≥ 25% in total Hocevar score
**Biological** *(assessed by IgG or RF)*	1	IgG: decrease ≥ 10%

		**or**<br>Rheumatoid factor: decrease ≥ 25%
	**Score**	**Change from baseline between groups**

STAR design 3A V13

**Domain**	**Level**	**Definition of response**
**Systemic activity**	Major	Decrease of clinESSDAI ≥ 5
**Patient reported outcome**	Major	Decrease of ESSPRI ≥ 1 point or ≥ 15%
**Lachrymal gland function**<br>*(assessed by Schirmer's test or Ocular Staining Score)*	Minor	Schirmer:<br>If abnormal score at baseline: increase ≥ 5 mm<br>If normal score at baseline: no change to abnormal<br>**or**<br>Ocular Staining Score:<br>If abnormal score at baseline: decrease ≥ 2 points<br>If normal score at baseline: no change to abnormal
**Salivary gland function**<br>*(assessed by unstimulated whole salivary flow or ultrasound)*	Minor	Unstimulated Whole Salivary Flow:<br>If score > 0 at baseline: increase ≥ 25%<br>If score is 0 at baseline: any increase in UWSF<br>**or**<br>Ultrasound:<br>Decrease ≥ 25% in total Hocevar score
**Biological**<br>*(assessed by IgG or RF)*	Minor	IgG: decrease ≥ 10%<br>**or**<br>Rheumatoid factor: decrease ≥ 25%
	**Responder**	**≥ 3 of 5 domains including at least 1 major domain**

STAR design 3A V17

**Domain**	**Level**	**Definition of response**
**Systemic activity**	Major	Decrease of clinESSDAI ≥ 5
**Patient reported outcome**<br>*(assessed by VAS of ocular dryness, oral dryness, fatigue and pain)*	Major	Decrease of 15% in at least 2 of 4 VAS
**Lachrymal gland function**<br>*(assessed by Schirmer's test or Ocular Staining Score)*	Minor	Schirmer:<br>If abnormal score at baseline: increase ≥ 5 mm<br>If normal score at baseline: no change to abnormal<br>**or**<br>Ocular Staining Score:<br>If abnormal score at baseline: decrease ≥ 2 points<br>If normal score at baseline: no change to abnormal

Seror R, *et al. Ann Rheum Dis* 2022; 81:979–989. doi: 10.1136/annrheumdis-2021-222054

**Salivary gland function**<br>*(assessed by unstimulated whole salivary flow or ultrasound)*	Minor	Unstimulated Whole Salivary Flow:<br>If score > 0 at baseline: increase ≥ 25%<br>If score is 0 at baseline: any increase in UWSF<br>**or**<br>Ultrasound:<br>Decrease ≥ 25% in total Hocevar score
**Biological**<br>*(assessed by IgG or RF)*	Minor	IgG: decrease ≥ 10%<br>**or**<br>Rheumatoid factor: decrease ≥ 25%
	**Responder**	**≥ 3 of 5 domains including at least 1 major domain**

STAR design 4 V2

**Domain**	**Definition of response**
**Systemic activity**	Improvement of clinESSDAI ≥ 20%
**Patient reported outcome**	Improvement of ESSPRI ≥ 20%
**Lachrymal gland function**<br>*(assessed by Schirmer's test or Ocular Staining Score)*	Improvement of Schirmer score ≥ 20%<br>**or**<br>Improvement of Ocular Staining Score ≥ 20%
**Salivary gland function**<br>*(assessed by unstimulated whole salivary flow or ultrasound)*	Improvement of Unstimulated Whole Salivary Flow score ≥ 20%<br>**or**<br>Improvement of total Hocevar score ≥ 20%
**Biological**<br>*(assessed by IgG or RF)*	Improvement in IgG level ≥ 20%<br>**or**<br>Improvement in Rheumatoid factor level ≥ 20%
**Responder**	**Improvement in ≥ 3 of 5 domains**

STAR design 3B threshold 6 V1

**Domain**	**Point**	**Definition of response**
**Systemic activity**	3	Decrease of clinESSDAI ≥ 3
**Patient reported outcome**	3	Decrease of ESSPRI ≥ 1 point or ≥ 15%
**Lachrymal gland function**<br>*(assessed by Schirmer's test or Ocular Staining Score)*	1	Schirmer:<br>If abnormal score at baseline: increase ≥ 5 mm<br>If normal score at baseline: no change to abnormal<br>**or**<br>Ocular Staining Score:<br>If abnormal score at baseline: decrease ≥ 2 points<br>If normal score at baseline: no change to abnormal

**Salivary gland function**<br>*(assessed by unstimulated whole salivary flow or ultrasound)*	1	Unstimulated Whole Salivary Flow:<br>If score > 0 at baseline: increase ≥ 25%<br>If score is 0 at baseline: any increase in UWSF<br>**or**<br>Ultrasound:<br>Decrease ≥ 25% in total Hocevar score
**Biological**<br>*(assessed by IgG or RF)*	1	IgG: decrease ≥ 10%<br>**or**<br>Rheumatoid factor: decrease ≥ 25%
	**Responder**	**≥ 6 points**

STAR design 3B threshold 6 V13

Domain	Point	Definition of response
**Systemic activity**	3	Decrease of clinESSDAI ≥ 5
**Patient reported outcome**	3	Decrease of ESSPRI ≥ 1 point or ≥ 15%
**Lachrymal gland function**<br>*(assessed by Schirmer's test or Ocular Staining Score)*	1	Schirmer:<br>If abnormal score at baseline: increase ≥ 5 mm<br>If normal score at baseline: no change to abnormal<br>**or**<br>Ocular Staining Score:<br>If abnormal score at baseline: decrease ≥ 2 points<br>If normal score at baseline: no change to abnormal
**Salivary gland function**<br>*(assessed by unstimulated whole salivary flow or ultrasound)*	1	Unstimulated Whole Salivary Flow:<br>If score > 0 at baseline: increase ≥ 25%<br>If score is 0 at baseline: any increase in UWSF<br>**or**<br>Ultrasound:<br>Decrease ≥ 25% in total Hocevar score
**Biological**<br>*(assessed by IgG or RF)*	1	IgG: decrease ≥ 10%<br>**or**<br>Rheumatoid factor: decrease ≥ 25%
	**Responder**	**≥ 6 points**

STAR design 4 V30

Domain	Definition of response
**Systemic activity**	Improvement of clinESSDAI ≥ 30%
**Patient reported outcome**	Improvement of ESSPRI ≥ 10%
**Lachrymal gland function**<br>*(assessed by Schirmer's test or Ocular Staining Score)*	Improvement of Schirmer score ≥ 30%<br>**or**<br>Improvement of Ocular Staining Score ≥ 30%

**Salivary gland function** *(assessed by unstimulated whole salivary flow or ultrasound)*	Improvement of Unstimulated Whole Salivary Flow score ≥ 30%<br>**or**<br>Improvement of total Hocevar score ≥ 30%	
**Biological** *(assessed by IgG or RF)*	Improvement in IgG level ≥ 10%<br>**or**<br>Improvement in Rheumatoid factor level ≥ 10%	
**Responder**		**Improvement in ≥ 3 of 5 domains**

STAR design 3B continuous V5

**Domain**	**Point**	**Definition of response**
**Systemic activity**	3	Decrease of clinESSDAI ≥ 3
**Patient reported outcome** *(assessed by VAS of ocular dryness, oral dryness, fatigue and pain)*	3	Decrease of 15% in at least 2 of 4 VAS
**Lachrymal gland function** *(assessed by Schirmer's test or Ocular Staining Score)*	1	Schirmer:<br>If abnormal score at baseline: increase ≥ 5 mm<br>If normal score at baseline: no change to abnormal<br>**or**<br>Ocular Staining Score:<br>If abnormal score at baseline: decrease ≥ 2 points<br>If normal score at baseline: no change to abnormal
**Salivary gland function** *(assessed by unstimulated whole salivary flow or ultrasound)*	1	Unstimulated Whole Salivary Flow:<br>If score > 0 at baseline: increase ≥ 25%<br>If score is 0 at baseline: any increase in UWSF<br>**or**<br>Ultrasound:<br>Decrease ≥ 25% in total Hocevar score
**Biological** *(assessed by IgG or RF)*	1	IgG: decrease ≥ 10%<br>**or**<br>Rheumatoid factor: decrease ≥ 25%
	**Score**	**Change from baseline between groups**

STAR design 3B continuous V17

**Domain**	**Point**	**Definition of response**
**Systemic activity**	3	Decrease of clinESSDAI ≥ 5
**Patient reported outcome** *(assessed by VAS of ocular dryness, oral dryness, fatigue and pain)*	3	Decrease of 15% in at least 2 of 4 VAS

**Lachrymal gland function**<br>*(assessed by Schirmer's test or Ocular Staining Score)*	1	Schirmer:<br>If abnormal score at baseline: increase ≥ 5 mm<br>If normal score at baseline: no change to abnormal<br>**or**<br>Ocular Staining Score:<br>If abnormal score at baseline: decrease ≥ 2 points<br>If normal score at baseline: no change to abnormal
**Salivary gland function**<br>*(assessed by unstimulated whole salivary flow or ultrasound)*	1	Unstimulated Whole Salivary Flow:<br>If score > 0 at baseline: increase ≥ 25%<br>If score is 0 at baseline: any increase in UWSF<br>**or**<br>Ultrasound:<br>Decrease ≥ 25% in total Hocevar score
**Biological**<br>*(assessed by IgG or RF)*	1	IgG: decrease ≥ 10%<br>**or**<br>Rheumatoid factor: decrease ≥ 25%
	**Score**	**Change from baseline between groups**

Seror R, et al. Ann Rheum Dis 2022; 81:979–989. doi: 10.1136/annrheumdis-2021-222054

Supplement 12: Design of the NECESSITY trial

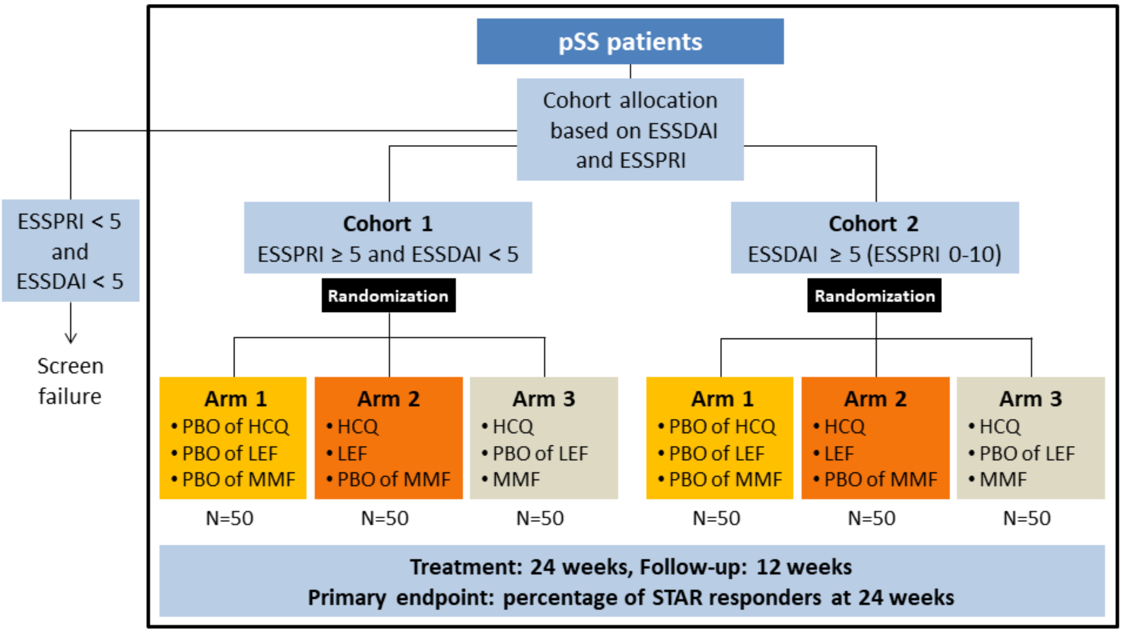

ESSDAI: EULAR Sjögren syndrome disease activity index, ESSPRI: EULAR Sjögren syndrome patient reported index,HCQ: hydroxychloroquine, LEF: leflunomide, MMF: mycophenolate mofetil, N: number, PBO: placebo, pSS: primary Sjögren syndrome

Seror R, *et al. Ann Rheum Dis* 2022; 81:979–989. doi: 10.1136/annrheumdis-2021-222054

Supplement 13: Letter of support from the NECESSITY Patient Advisory Group

Paris, 24th of November 2021

Letter of support for the novel STAR outcome.

Sjögren's is a complex systemic autoimmune disease that affects the whole body. Along with symptoms of extensive dryness, other serious complications include profound fatigue, chronic pain, major organ involvement, neuropathies and lymphomas.

There are currently no available medicines to halt or reverse the progression of this debilitating disease. Some treatments are available to alleviate some of the symptoms, but there are many unmet needs. Patients' quality of life is dramatically impaired. Moreover, there is a distinct lack of knowledge of the disease among GPs which frequently results in an incorrect and/or delayed diagnosis. Patients are affected physically, mentally, emotionally, socially and financially.

The burden on individual patients and their families is significant and the burden on society as a whole must not be ignored as individual sufferers frequently become unable to work and play an active role in society as a result of the impact of their Sjögren's.

The need for effective treatment is urgent. However, the heterogeneous nature of the disease has, thus far, made it difficult to accurately access the effectiveness in clinical trials of any proposed new product using existing indices.

This multi-national collaboration NECESSITY, (stands for New Clinical Endpoints in primary Sjögren's Syndrome: an Interventional Trial based on Stratifying Patients", NECESSITY (necessity-h2020.eu)) has been designed to improve on the currently most-used disease activity index, ESSDAI. AFGS (Association Française du Gougerot-Sjögren), a patient association partner of the NECESSITY project, is coordinating the interaction with patients and has created a Patient Advisory Group (PAG) Patient Advisory Group - NECESSITY (necessity-h2020.eu). The PAG independently provides guidance from the patient perspective on the project's activities such as the development of the novel outcome STAR. In particular, 20 patients contributed to the identification of the domains and measurements to include in STAR. Some patients gave their opinion at the time of defining the final version of STAR. The use of the new more appropriate outcome measure identified by the NECESSITY project should improve the definition of clinical trial results in Sjögren's. **This improved definition may lead to the development and approval of the first systemic therapy for Sjögren's which will have a dramatic impact on the lives of millions of Sjögren's patients around the world**. The PAG strongly supports the newly developed STAR outcome.

Association Française du Gougerot Sjögren et des Syndromes Secs
Association à but non lucratif, fondée en 1990, reconnue d'utilité publique
Adresse postale : AFGS - 9, rue du Château 67540 OSTWALD
Tél 03 88 28 55 99 - Email : contact@afgs-syndromes-secs.org

With hope for a better future for Sjögren's patients,

Maggy Pincemin, AFGS
On behalf of the **Necessity Patient Advisory Group**

Association Française du Gougerot-Sjögren et des Syndromes Secs (France)

Diagnosegruppe Sjøgrens Syndrom (Norway)

Asociación Española de Síndrome de Sjögren (Spain)

Nationale Vereniging Sjögrenpatiënten (The Netherlands)

British Sjögren's Syndrome Association (The United Kingdom)

Sjögren's Foundation (The United States of America)

Association Française du Gougerot Sjögren et des Syndromes Secs
Association à but non lucratif, fondée en 1990, reconnue d'utilité publique
Adresse postale : AFGS - 9, rue du Château 67540 OSTWALD
Tél 03 88 28 55 99 - Email : contact@afgs-syndromes-secs.org
